# Stochastic proximal gradient methods for nonconvex problems in Hilbert spaces

**DOI:** 10.1007/s10589-020-00259-y

**Published:** 2021-01-12

**Authors:** Caroline Geiersbach, Teresa Scarinci

**Affiliations:** 1 Weierstrass Institute, Mohrenstrasse 39, 10117 Berlin, Germany; 2 Department of Information Engineering, Computer Science and Mathematics, University of L’Aquila, Via Vetoio - Loc. Coppito, 67010 L’Aquila, Italy

**Keywords:** Stochastic programming, Nonsmooth and nonconvex optimization, Differential inclusions, Mathematical programming methods, Partial differential equations with randomness, Optimal control problems involving partial differential equations

## Abstract

For finite-dimensional problems, stochastic approximation methods have long been used to solve stochastic optimization problems. Their application to infinite-dimensional problems is less understood, particularly for nonconvex objectives. This paper presents convergence results for the stochastic proximal gradient method applied to Hilbert spaces, motivated by optimization problems with partial differential equation (PDE) constraints with random inputs and coefficients. We study stochastic algorithms for nonconvex and nonsmooth problems, where the nonsmooth part is convex and the nonconvex part is the expectation, which is assumed to have a Lipschitz continuous gradient. The optimization variable is an element of a Hilbert space. We show almost sure convergence of strong limit points of the random sequence generated by the algorithm to stationary points. We demonstrate the stochastic proximal gradient algorithm on a tracking-type functional with a $L^1$-penalty term constrained by a semilinear PDE and box constraints, where input terms and coefficients are subject to uncertainty. We verify conditions for ensuring convergence of the algorithm and show a simulation.

## Introduction

In this paper, we focus on stochastic approximation methods for solving a stochastic optimization problem on a Hilbert space *H* of the form

P $$\begin{aligned} \min _{u \in H} \{f(u) = j(u) + h(u) \}, \end{aligned}$$

where the expectation $j(u) = \mathbb {E}[J(u, \xi )]$ is generally nonconvex with a Lipschitz continuous gradient and *h* is a proper, lower semicontinuous, and convex function that is generally nonsmooth.

Our work is motivated by applications to PDE-constrained optimization under uncertainty, where a nonlinear PDE constraint can lead to an objective function that is nonconvex with respect to the Hilbert-valued variable. To handle the (potentially infinite-dimensional) expectation, algorithmic approaches for solving such problems involve either some discretization of the stochastic space or an ensemble-based approach with sampling or carefully chosen quadrature points. Stochastic discretization includes polynomial chaos and the stochastic Galerkin method; cf. [24, 30, 34, 47]. For ensemble-based methods, the simplest method is sample average approximation (SAA), where the original problem is replaced by a proxy problem with a fixed set of samples, which can then be solved using a deterministic solver. A number of standard improvements to Monte Carlo sampling have been applied to optimal control problems in, e.g., [1, 54]. Another ensemble-based approach is the stochastic collocation method, which has been used in optimal control problems in e.g. [47, 51]. Sparse-tensor discretization has been used for optimal control problems in, for instance, [28, 29].

The approach we use is an ensemble-based approach called stochastic approximation, which is fundamentally different in the sense that sampling takes place dynamically as part of the optimization procedure, leading to an algorithm with low complexity and computational effort when compared to other approaches. Stochastic approximation originated in a groundbreaking paper by [45], where an iterative method to find the root of an unknown function using noisy estimates was proposed. The authors of [25] used this idea to solve a regression problem using finite differences subject to noise. Algorithms of this kind, with bias in addition to stochastic noise, are sometimes called stochastic quasi-gradient methods; see, e.g., [17, 53]. Basic versions of these algorithms rely on positive step sizes $t_n$ of the form $\sum _{n=1}^\infty t_n = \infty$ and $\sum _{n=1}^\infty t_n ^2 < \infty$. The (almost sure) asymptotic convergence of stochastic approximation algorithms for convex problems is classical in finite dimensions; we refer to the texts by [16, 33].

There have been a number of contributions with proofs of convergence of the stochastic gradient method for unconstrained nonconvex problems; see [6, 7, 49, 56]. Fewer results exist for constrained and/or nonsmooth nonconvex problems. A randomized stochastic algorithm was proposed by Ghadimi et al. [21]; this scheme involves running a stochastic approximation process and randomly choosing an iterate from the generated sequence. There have been some contributions involving constant step sizes with increasing sampling; see [35, 44]. Convergence of projection-type methods for nonconvex problems was shown in [32] and for prox-type methods by Davis et al. [13].

As far as stochastic approximation on function spaces is concerned, many contributions were motivated by applications with nonparametric statistics. Perhaps the oldest example is from [55]. Goldstein [22] studied an infinite-dimensional version of the Kiefer–Wolfowitz procedure. A significant contribution for unconstrained problems was by Yin and Zhu [58]. Projection-type methods were studied by [3, 10, 12, 40].

In this paper, we prove convergence results for nonconvex and nonsmooth problems in Hilbert spaces. We present convergence analysis that is based on the recent contributions in [13, 35]. Applications of the stochastic gradient method to PDE-constrained optimization have already been explored by [19, 37]. In these works, however, convexity of the objective function is assumed, leaving the question of convergence in the more general case entirely open. We close that gap by making the following contributions:

- For an objective function that is the sum of a smooth, generally nonconvex expectation and a convex, nonsmooth term, we prove that strong accumulation points of iterates generated by the method are stationary points.
- We show that convergence holds even in the presence of systematic additive bias, which is relevant for the application in mind.
- We demonstrate the method on an application to PDE-constrained optimization under uncertainty and verify conditions for convergence.

The paper is organized as follows. In Sect. 2, notation and background is given. Convergence of two related algorithms is proven in Sect. 3. In Sect. 4, we introduce a problem in PDE-constrained optimization under uncertainty, where coefficients in the semilinear PDE constraint are subject to uncertainty. The problem is shown to satisfy conditions for convergence, and numerical experiments demonstrate the method. We finish the paper with closing remarks in Sect. 5.

## Notation and background

We recall some notation and background from convex analysis and stochastic processes; see [4, 11, 38, 43].

Let *H* be a Hilbert space with the scalar product $\langle \cdot , \cdot \rangle$ and norm $\Vert \cdot \Vert$. The symbols $\rightarrow$ and $\rightharpoonup$ denote strong and weak convergence, respectively. The set of proper, convex, and lower semicontinuous functions $h:H \rightarrow (-\infty , \infty ]$ is denoted by $\varGamma _0(H)$. Given a function $h \in \varGamma _0(H)$ and $t > 0$, the proximity operator $\text {prox}_{th}:H \rightarrow H$ is given by

$$\begin{aligned} \text {prox}_{t h}(u) := \underset{v\in H}{\mathrm{arg\,min}} \left( h(v) + \frac{1}{2t} \Vert v-u\Vert ^2\right) . \end{aligned}$$

We recall that for a proper function $h:H \rightarrow (-\infty , \infty ]$, the subdifferential (in the sense of convex analysis) is the set-valued operator

$$\begin{aligned} \partial h: H \rightrightarrows H: u \mapsto \{ v \in H: \langle y - u, v \rangle + h(u) \le h(y) \quad \forall y \in H \}. \end{aligned}$$

For any $h \in \varGamma _0(H)$, the subdifferential $\partial h$ is maximally monotone. The domain of *h* is denoted by $\mathrm{dom}(h)$. The indicator function of a set *C* is denoted by $\delta _C$, where $\delta _C(u) = 0$ if $u \in C$ and $\delta _C(u) = \infty$ otherwise. The sum of two sets *A* and *B* with $\lambda \in \mathbb {R}$ is given by $A+\lambda B:=\{ a+\lambda b: a \in A, b \in B\}.$ The distance of a point *u* to a nonempty, closed set *A* is denoted by $d(u,A):=\inf _{a \in A} \Vert u-a\Vert$ and the diameter of *A* is denoted by the symbol $\text {diam}(A):=\sup _{u, v \in A} \Vert u - v \Vert$. For a nonempty and convex set *C*, the normal cone $N_C(u)$ at $u \in C$ is defined by

$$\begin{aligned} N_C(u):= \{ z \in H: \langle z, w-u \rangle \le 0, \quad \forall w \in C\}. \end{aligned}$$

We set $N_C(u) := \emptyset$ if $u \notin C$. We recall that $\partial \delta _C(u) = N_C(u)$ for all $u \in C$. If $h_1,h_2 \in \varGamma _0(H)$ and $\mathrm{dom} (h_2) = H$, then $\partial [h_1(u) + h_2(u)] = \partial h_1(u) +\partial h_2(u)$. If *h* is proper and $\mathrm{dom} (h)$, then $\partial h(u)$ is closed and convex. We recall that the graph of $\partial h$ for a function $h \in \varGamma _0(H)$, given by the set $\mathrm{gra} (\partial h) = \{ (u,\partial h(u)): u \in H\}$, is sequentially closed in the strong-to-weak topology, meaning that for $u_n \rightarrow u$, $\zeta _n \in \partial h(u_n)$, and $\zeta _n \rightharpoonup \zeta$, it follows that $\zeta \in \partial h(u)$. The normal cone $N_C(u)$ is strong-to-weak sequentially closed if *C* is convex.

Throughout, $(\varOmega , \mathcal {F}, \mathbb {P})$ will denote a probability space, where $\varOmega$ represents the sample space, $\mathcal {F} \subset 2^{\varOmega }$ is the $\sigma$-algebra of events on the power set of $\varOmega$, denoted by $2^{\varOmega }$, and $\mathbb {P}:\varOmega \rightarrow [0,1]$ is a probability measure. Given a random vector $\xi :\varOmega \rightarrow \varXi \subset \mathbb {R}^m$, we write $\xi \in \varXi$ to denote a realization of the random vector. The operator $\mathbb {E}[\cdot ]$ denotes the expectation with respect to this distribution; for a parametrized functional $J: H \times \varXi \rightarrow \mathbb {R}$, this is defined as the integral over all elements in $\varOmega$, i.e.,

$$\begin{aligned} \mathbb {E}[J(u,\xi )] = \int _\varOmega J(u,\xi (\omega )) \,\mathrm {d}\mathbb {P}(\omega ). \end{aligned}$$

A filtration is a sequence $\{ \mathcal {F}_n\}$ of sub-$\sigma$-algebras of $\mathcal {F}$ such that $\mathcal {F}_1 \subset \mathcal {F}_2 \subset \cdots \subset \mathcal {F}.$ We define a discrete *H*-valued stochastic process as a collection of *H*-valued random variables indexed by *n*, in other words, the set $\{ \beta _n: \varOmega \rightarrow H \, \vert \, n \in \mathbb {N}\}.$ The stochastic process is said to be adapted to a filtration $\{ \mathcal {F}_n \}$ if and only if $\beta _n$ is $\mathcal {F}_n$-measurable for all *n*. The natural filtration is the filtration generated by the sequence $\{\beta _n\}$ and is given by $\mathcal {F}_n = \sigma (\{\beta _1, \dots ,\beta _n\})$.^1^ If for an event $F \in \mathcal {F}$ it holds that $\mathbb {P}(F) = 1$, or equivalently, $\mathbb {P}(\varOmega \backslash F) = 0$, we say *F* occurs almost surely (a.s.). Sometimes we also say that such an event occurs with probability one. A sequence of random variables $\{\beta _n\}$ is said to converge almost surely to a random variable $\beta$ if and only if

$$\begin{aligned} \mathbb {P}\left( \left\{ \omega \in \varOmega : \lim _{n \rightarrow \infty } \beta _n(\omega ) = \beta (\omega ) \right\} \right) = 1. \end{aligned}$$

For an integrable random variable $\beta :\varOmega \rightarrow \mathbb {R}$, the conditional expectation is denoted by $\mathbb {E}[\beta | \mathcal {F}_n]$, which is itself a random variable that is $\mathcal {F}_n$-measurable and which satisfies $\int _A \mathbb {E}[\beta | \mathcal {F}_n](\omega ) \,\mathrm {d}\mathbb {P}(\omega ) = \int _A \beta (\omega ) \,\mathrm {d}\mathbb {P}(\omega )$ for all $A \in \mathcal {F}_n$. Almost sure convergence of *H*-valued stochastic processes and conditional expectation are defined analogously.

Given a random operator $F:X \times \varOmega \rightarrow Y$, where *X* and *Y* are Banach spaces, we will sometimes use the notation $F_\omega :=F(\cdot , \omega ):X \rightarrow Y$ for a fixed (but arbitrary) $\omega \in \varOmega$. For a Banach space $(X,\Vert \cdot \Vert _X)$, the Bochner space $L^p(\varOmega ,X)$ is the set of all (equivalence classes of) strongly measurable functions $u:\varOmega \rightarrow X$ having finite norm, where the norm is defined by

$$\begin{aligned} \Vert u \Vert _{L^p(\varOmega ,X)}:= {\left\{ \begin{array}{ll} (\int _\varOmega \Vert u(\omega ) \Vert _X^p \,\mathrm {d}\mathbb {P}(\omega ))^{1/p}, \quad &p < \infty \\ {{\,\mathrm{ess\,sup}\,}}_{\omega \in \varOmega } \Vert u(\omega ) \Vert _X, \quad &p=\infty \end{array}\right. }. \end{aligned}$$

A sequence $\{\beta _n\}$ in $L^1(\varOmega , X)$ is called a martingale if a filtration $\{ \mathcal {F}_n\}$ exists such that $\beta _n$ is $\mathcal {F}_n$-measurable and $\mathbb {E}[\beta _{n+1}|\mathcal {F}_n] = \beta _{n}$ is satisfied for all *n*.

For an open subset *U* of a Banach space *X* and a function $J_\omega :U \rightarrow \mathbb {R}$, we denote the Gâteaux derivative at $u \in U$ in the direction $v \in X$ by $dJ_\omega (u; v).$ The Fréchet derivative at *u* is denoted by $J_\omega ':U \rightarrow \mathcal {L}(X,\mathbb {R})$, where $\mathcal {L}(X,\mathbb {R})$ is the set of bounded and linear operators mapping *X* to $\mathbb {R}$. We recall this is none other than the dual space $X^*$ and we denote the dual pairing by $\langle \cdot , \cdot \rangle _{X^*,X}$. For an open subset *U* of a Hilbert space *H* and a Fréchet differentiable function $j:U \rightarrow \mathbb {R}$, the gradient $\nabla j:U \rightarrow H$ is the Riesz representation of $j':U \rightarrow H^*$, i.e., it satisfies $\langle \nabla j(u), v \rangle = \langle j'(u),v \rangle _{H^*,H}$ for all $u \in U$ and $v \in H.$ In Hilbert spaces, the Riesz representation relates elements of the dual space to the Hilbert space itself, allowing us to drop the dual pairing notation and use simply $\langle \cdot , \cdot \rangle$.

The notation $C_L^{1,1}(U)$ is used to denote the set of continuously differentiable functions on $U \subset H$ with an *L*-Lipschitz gradient, meaning $\Vert \nabla j(u) - \nabla j(v) \Vert \le L \Vert u - v \Vert$ is satisfied for all $u,v \in U.$ The following lemma gives a classical Taylor estimate for such functions.

### Lemma 2.1

*Suppose* $j \in C_L^{1,1}(U)$, $U\subset H$ *open and convex. Then for all* $u, v \in U$,

$$\begin{aligned} j(v) + \langle \nabla j(v), u-v\rangle - \frac{L}{2} \Vert u - v \Vert ^2 \le j(u) \le j(v) + \langle \nabla j(v), u-v\rangle + \frac{L}{2} \Vert u-v\Vert ^{2}. \end{aligned}$$

## Asymptotic convergence results

In this section, we show asymptotic convergence results for two variants of the stochastic proximal gradient method in Hilbert spaces for solving Problem (P). Let $G:H \times \varXi \rightarrow H$ be a parametrized operator (the *stochastic gradient*) approximating (in a sense to be specified later) the gradient $\nabla j:H \rightarrow H$ and let $t_n$ be a positive step size. Both algorithms in this section will share the basic iterative form

$$\begin{aligned} u_{n+1} := \text {prox}_{t_n h}(u_n - t_n G(u_n,\xi _n)), \end{aligned}$$

where *h* is the nonsmooth term from Problem (P). The following assumptions will be in force in all sections.

### Assumption 3.1

Let $\{\mathcal {F}_n \}$ be a filtration and let $\{ u_n\}$ and $\{G(u_n,\xi _n)\}$ be sequences of iterates and stochastic gradients. We assume

(i) The sequence $\{ u_n\}$ is a.s. contained in a bounded set $V \subset H$ and $u_n$ is adapted to $\mathcal {F}_n$ for all *n*.
(ii) On an open and convex set *U* such that $V \subset U \subset H$, the expectation $j\in C_L^{1,1}(U)$ is bounded below.
(iii) For all *n*, the *H*-valued random variable $r_n := \mathbb {E}[G(u_n,\xi _n) | \mathcal {F}_n] - \nabla j(u_n)$ is adapted to $\mathcal {F}_n$ and for $K_n:={{\,\mathrm{ess\,sup}\,}}_{\omega \in \varOmega } \Vert r_n(\omega )\Vert$, $\sum _{n=1}^\infty t_n K_n< \infty$ and $\sup _{n} K_n<\infty$ are satisfied.
(iv) For all *n*, $\mathfrak {w}_n := G(u_n, \xi _n) - \mathbb {E}[G(u_n, \xi _n) | \mathcal {F}_n]$ is an *H*-valued random variable.

### Remark 3.2

The assumption that the sequence $\{ u_n\}$ stays bounded with probability one is by no means automatically fulfilled, but can be verified or enforced in different ways. We refer to [6, Section 5.2] and [13, Section 6.1] for conditions on the function, constraint set, and/or regularizers that ensure boundedness of iterates. The conditions in Assumption 3.1 allow for additive bias $r_n$ in the stochastic gradient in addition to zero-mean error $\mathfrak {w}_n$. The requirement that $u_n$ and $r_n$ are adapted to $\mathcal {F}_n$ is automatically fulfilled if $\{ \mathcal {F}_n\}$ is chosen to be the natural filtration generated by $\{\xi _1, \dots , \xi _n \}$. Together, Assumption 3.1(iii) and Assumption 3.1(iv) imply

$$\begin{aligned} G(u_n,\xi _n) = \nabla j(u_n) + r_n + \mathfrak {w}_n \end{aligned}$$

and $\mathbb {E}[\mathfrak {w}_n | \mathcal {F}_n] = 0.$ Notice that a single realization $\xi _n \in \varXi$ can be replaced by $m_n$ independently drawn realizations $\xi _{n}^1, \dots , \xi _{n}^{m_n} \in \varXi$ since

$$\begin{aligned} \mathbb {E}[G(u_n,\xi _n)|\mathcal {F}_n] =\frac{1}{m_n} \mathbb {E}\left[ \sum _{i=1}^{m_n} G(u_n,\xi _{n}^{i}) |\mathcal {F}_n \right] . \end{aligned}$$

This set of $m_n$ samples is sometimes called a “batch”; batches clearly reduce the variance of the stochastic gradient.

The result in Sect. 3.1 shows asymptotic convergence of the proximal gradient method with constant step sizes and increasing sampling. In Sect. 3.2, we switch to the versatile ordinary differential equation (ODE) method to prove convergence of the stochastic proximal gradient method with decreasing step sizes. We emphasize that the convergence results generalize existing convergence theory from the finite-dimensional case. Our analysis includes convergence in possibly infinite-dimensional Hilbert spaces. Additionally, we allow for stochastic gradients subject to additive bias, which is not covered by existing results. This theory can be used to develop mesh refinement strategies in applications with PDEs [20].

### Variance-reduced stochastic proximal gradient method

In this section, we show under what conditions the variance-reduced stochastic proximal gradient method converges to stationary points for Problem (P). With $\xi _n = (\xi _{n}^1,\dots , \xi _{n}^{m_n}),$ the stochastic gradient is given by the average

$$\begin{aligned} G(u_n,\xi _n) = \frac{\sum _{i=1}^{m_n}G(u_n,\xi _{n}^i)}{m_n} \end{aligned}$$

over an *increasing* number of samples $m_n$. The algorithm is presented below, which uses constant step sizes $t_n \equiv t$ depending on the Lipschitz constant *L* from Assumption 3.1(ii). 
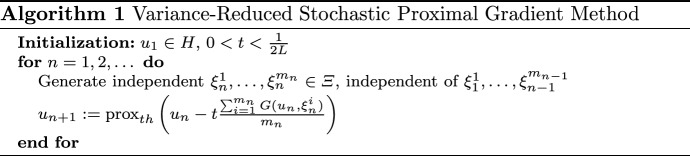


#### Remark 3.3

If $h(u)=\delta _C(u)$ and $\pi _C$ denotes the projection onto *C*, then the algorithm reduces to $u_{n+1}:=\pi _C\left( u_n - t \frac{\sum _{i=1}^{m_n}G(u_n,\xi _{n}^i)}{m_n}\right) ,$ i.e., the variance-reduced projected stochastic gradient method.

In addition to Assumption 3.1, the following assumptions will be in force in this section.

#### Assumption 3.4

Let $\{ u_n\}$ and $\{G(u_n,\xi _n)\}$ be generated by Algorithm 1. We assume

(i) The function *h* satisfies $h \in \varGamma _0(H)$.
(ii) For all *n*,
  $$\begin{aligned} w_n : = \frac{\sum _{i=1}^{m_n}G(u_n,\xi _{n}^i)}{m_n} - \nabla j(u_n) \end{aligned}$$
  is an *H*-valued random variable and there exists an $M \ge 0$ such that $\mathbb {E}[\Vert w_n\Vert ^2 | \mathcal {F}_n] \le \frac{M}{m_n}$ and $\sum _{n=1}^\infty \tfrac{1}{m_n} < \infty$.

#### Remark 3.5

We use assumptions similar to those found in [35], but we do not require the effective domain of *h* to be bounded; we instead use boundedness of the iterates by Assumption 3.1(i). Notice that $w_n = r_n + \mathfrak {w}_n$ from Assumption 3.1(iv), hence Assumption 3.4(ii) also provides a condition on the rate at which $r_n$ and $\mathfrak {w}_n$ must decay.

For the convergence result, we need the following lemma [46].

#### Lemma 3.6

(Robbins–Siegmund) *Assume that* $\{\mathcal {F}_n\}$ *is a filtration and* $v_n$, $a_n$, $b_n$, $c_n$ *are nonnegative random variables adapted to* $\mathcal {F}_n.$ *If*

$$\begin{aligned} \mathbb {E}[v_{n+1} | \mathcal {F}_n] \le v_n(1+a_n)+ b_n-c_n \quad \text {a.s.} \end{aligned}$$

*and* $\sum _{n=1}^\infty a_n< \infty , \sum _{n=1}^\infty b_n < \infty$ *a.s., then with probability one,* $\{v_n\}$ *is convergent and* $\sum _{n=1}^\infty c_n < \infty$.

To show convergence, we first present a technical lemma.

#### Lemma 3.7

*Let* $u\in U$ *and* $t>0$. *Suppose* $v:=\text {prox}_{t h}(u-t g) \in U$ *for a given* $g \in H$. *Then for any* $z \in U$,

3.1 $$\begin{aligned} \begin{aligned} f(v)&\le f(z) + \langle v-z, \nabla j(u) - g\rangle + \left( \frac{L}{2}- \frac{1}{2t}\right) \Vert v-u \Vert ^2\\&\quad \quad + \left( \frac{L}{2} + \frac{1}{2t}\right) \Vert z-u \Vert ^2 - \frac{1}{2t} \Vert v-z \Vert ^2. \end{aligned} \end{aligned}$$

#### Proof

We first claim that for all $y,z\in H$, $t>0$ and $p=\text {prox}_{th}(y)$,

3.2 $$\begin{aligned} h(p) + \frac{1}{2t} \Vert p-y \Vert ^2 \le h(z) + \frac{1}{2t} \Vert z-y \Vert ^2 - \frac{1}{2t} \Vert p-z \Vert ^2. \end{aligned}$$

This follows by definition of the $\text {prox}$ operator. Indeed, for $t > 0$, $p = \text {prox}_{t h}(y)$ if and only if for all $z \in H$,

3.3 $$\begin{aligned} h(z) \ge h(p) + \frac{1}{t} \langle y -p, z-p \rangle . \end{aligned}$$

It is straightforward to verify the following equality (the law of cosines)

3.4 $$\begin{aligned} \Vert z - y \Vert ^2 = \Vert z-p\Vert ^2 + \Vert p-y\Vert ^2 - 2 \langle y-p, z-p\rangle . \end{aligned}$$

Multiplying (3.4) by $\tfrac{1}{2t}$ and adding it to (3.3), we get (3.2). Now, since $j\in C^{1,1}_L(U)$, it follows by Lemma 2.1 for $u,v,z\in U$ that

3.5 $$\begin{aligned} j(v)&\le j(u) + \langle \nabla j(u), v-u \rangle + \frac{L}{2} \Vert v - u \Vert ^2, \end{aligned}$$

3.6 $$\begin{aligned} j(u)&\le j(z) + \langle \nabla j(u), u-z \rangle + \frac{L}{2} \Vert z - u \Vert ^2. \end{aligned}$$

Combining (3.5) and (3.6), we get

3.7 $$\begin{aligned} j(v) \le j(z) + \langle \nabla j(u), v-z \rangle + \frac{L}{2} \Vert v - u \Vert ^2 + \frac{L}{2} \Vert z - u \Vert ^2. \end{aligned}$$

Now, by (3.2) applied to $v = \text {prox}_{th}(u-tg)$,

$$\begin{aligned} h(v) + \frac{1}{2t} \Vert v-(u-tg)\Vert ^2&\le h(z) +\frac{1}{2t} \Vert z-(u-tg)\Vert ^2 -\frac{1}{2t} \Vert v-z\Vert ^2 \end{aligned}$$

if and only if

3.8 $$\begin{aligned} \begin{aligned}&h(v) + \frac{1}{2t} \Vert v-u \Vert ^2 + \langle v-u,g\rangle \\&\quad \le h(z)+\frac{1}{2t} \Vert z-u\Vert ^2+\langle z-u,g\rangle -\frac{1}{2t} \Vert v-z\Vert ^2. \end{aligned} \end{aligned}$$

Finally, adding (3.7) and (3.8), and using that $f = j+h$, we get (3.1). $\square$

In the following, we define

3.9 $$\begin{aligned} \bar{u}_{n+1} := \text {prox}_{t h}(u_n - t \nabla j(u_n) ) \end{aligned}$$

as the iterate at $n+1$ if the true gradient were used.

#### Lemma 3.8

*For all* *n*,

3.10 $$\begin{aligned} \mathbb {E}[f(u_{n+1}) |\mathcal {F}_n] \le f(u_n) - \left( \frac{1}{2t} - L \right) \Vert \bar{u}_{n+1} - u_n \Vert ^2 + \frac{t}{2} \mathbb {E}[ \Vert w_n \Vert ^2 | \mathcal {F}_n] \quad \text{a.s.} \end{aligned}$$

#### Proof

Using Lemma 3.7 with $v = \bar{u}_{n+1}$, $u=z=u_n$, and $g = \nabla j(u_n)$, we have

3.11 $$\begin{aligned} f(\bar{u}_{n+1}) \le f(u_n) + \left( \frac{L}{2} - \frac{1}{t}\right) \Vert \bar{u}_{n+1} - u_n \Vert ^2. \end{aligned}$$

Again using Lemma 3.7, with $v=u_{n+1}$, $z=\bar{u}_{n+1}$, $u=u_n$, and $g = \nabla j(u_n) + w_n$, we get

3.12 $$\begin{aligned} \begin{aligned} f(u_{n+1})&\le f(\bar{u}_{n+1}) - \langle u_{n+1}-\bar{u}_{n+1}, w_n\rangle + \left( \frac{L}{2} - \frac{1}{2t}\right) \Vert u_{n+1} - u_n \Vert ^2 \\&\quad + \left( \frac{L}{2} + \frac{1}{2t}\right) \Vert \bar{u}_{n+1} - u_n \Vert ^2 - \frac{1}{2t} \Vert u_{n+1}-\bar{u}_{n+1}\Vert ^2. \end{aligned} \end{aligned}$$

By Young’s inequality, $\langle u_{n+1}-\bar{u}_{n+1}, w_n\rangle \le \frac{1}{2t}\Vert u_{n+1}-\bar{u}_{n+1} \Vert ^2 + \frac{t}{2} \Vert w_n \Vert ^2,$ so combining (3.11) and (3.12), we obtain since $0< t < \tfrac{1}{2L}$ that

3.13 $$\begin{aligned} \begin{aligned} f(u_{n+1})&\le f(u_n) + \left( L-\frac{1}{2t} \right) \Vert \bar{u}_{n+1}-u_{n}\Vert ^2 + \left( \frac{L}{2}-\frac{1}{2t} \right) \Vert u_{n+1}-u_{n}\Vert ^2 \\&\quad + \frac{t}{2} \Vert w_n \Vert ^2 \\&\le f(u_n) + \left( L-\frac{1}{2t} \right) \Vert \bar{u}_{n+1}-u_{n}\Vert ^2 + \frac{t}{2} \Vert w_n \Vert ^2. \end{aligned} \end{aligned}$$

Taking conditional expectation on both sides of (3.13), and noting that $\bar{u}_{n+1}$ is $\mathcal {F}_n$-measurable by $\mathcal {F}_n$-measurability of $u_n$, we get (3.10). $\square$

#### Remark 3.9

Any bounded sequence $\{ u_n\}$ in *H* contains a weakly convergent subsequence $\{ u_{n_k}\}$ such that $u_{n_k} \rightharpoonup u$ for a $u \in H.$ Generally this convergence is not strong, so we cannot conclude from $\Vert \bar{u}_{n+1}-u_{n}\Vert ^2 \rightarrow 0$ that there exists a $\tilde{u}$ such that, for a subsequence $\{ u_{n_k}\}$, $\lim _{k \rightarrow \infty } \bar{u}_{n_k+1} = \lim _{k \rightarrow \infty } u_{n_k} = \tilde{u}.$ Therefore, to obtain convergence to stationary points, we will assume that $\{ u_n\}$ has a strongly convergent subsequence.

We are ready to state the convergence result for sequences generated by Algorithm 1.

#### Theorem 3.10

*Let Assumptions* 3.1*and* 3.4*hold. Then*

1. *The sequence* $\{ f(u_n)\}$ *converges a.s.*
2. *The sequence* $\{ \Vert \bar{u}_{n+1} - u_n \Vert \}$ *converges to zero a.s.*
3. *Every strong accumulation point of* $\{ u_n\}$ *is a stationary point with probability one.*

#### Proof

The sequence $\{ u_n\}$ is contained in a bounded set *V* by Assumption 3.1(i). By Assumption 3.4(i), $h \in \varGamma _0(H)$ must therefore be bounded below on *V* [4, Corollary 9.20]; *j* is bounded below by Assumption 3.1(ii). W.l.o.g. we can thus assume $f \ge 0$. Since $\frac{1}{2t} > L$ and $\sum _{n=1}^\infty \mathbb {E}[\Vert w_n \Vert ^2 |\mathcal {F}_n] < \infty$ by Assumption 3.4(ii), we can apply Lemma 3.6 to (3.10) to conclude that $f(u_n)$ converges almost surely. The second statement follows immediately, since by Lemma 3.6,

3.14 $$\begin{aligned} \sum _{n=1}^\infty \Vert \bar{u}_{n+1} - u_n \Vert ^2 < \infty \quad \text {a.s.}, \end{aligned}$$

which implies that for almost every sample path, $\lim _{n \rightarrow \infty } \Vert \bar{u}_{n+1} - u_n \Vert ^2 = 0.$

For the third statement, we have that there exists a subsequence $\{ u_{n_k}\}$ such that $u_{n_k} \rightarrow u$. We argue that then $\bar{u}_{n_k+1} \rightarrow u$. Since $\{ \bar{u}_{n_k+1}\}$ is bounded, there exists a weak limit point $\tilde{u}$ (potentially on a subsequence with the same labeling). Then, using weak lower semicontinuity of the norm as well as the rule $\langle a_n, b_n \rangle \rightarrow \langle a,b\rangle$ for $a_n \rightharpoonup a$ and $b_n \rightarrow b$,

$$\begin{aligned} 0& = \lim _{k \rightarrow \infty } \Vert \bar{u}_{n_k+1} - u_{n_k}\Vert ^2 = \lim _{k \rightarrow \infty } \Vert \bar{u}_{n_k+1} \Vert ^2 - 2 \langle \bar{u}_{n_k+1}, u_{n_k} \rangle + \Vert u_{n_k} \Vert ^2\\& = \liminf _{k \rightarrow \infty } \Vert \bar{u}_{n_k+1} \Vert ^2 - 2 \langle \bar{u}_{n_k+1}, u_{n_k} \rangle + \Vert u_{n_k} \Vert ^2 \\&\ge \Vert \tilde{u} \Vert ^2 - 2 \langle \tilde{u}, u \rangle + \Vert u \Vert ^2 = \Vert \tilde{u} - u \Vert ^2 \ge 0, \end{aligned}$$

implying $u=\tilde{u}.$ It follows $\bar{u}_{n_k+1} \rightarrow u$ by assuming $\lim _{k \rightarrow \infty } \Vert \bar{u}_{n_k+1} \Vert ^2 \ne \Vert u \Vert ^2$ and arriving at a contradiction. Now, by definition of the $\text {prox}$ operator,

$$\begin{aligned} \bar{u}_{n_k+1}& = \text {prox}_{t h}(u_{n_k} - t \nabla j(u_{n_k}) )\\& = \underset{v \in H}{\mathrm{arg\,min}} \Big \lbrace h(v) + \frac{1}{2t} \Vert v- u_{n_k} + t \nabla j(u_{n_k}) \Vert ^2 \Big \rbrace \\& = \underset{v \in H}{\mathrm{arg\,min}} \Big \lbrace h(v) + \langle \nabla j(u_{n_k}), v \rangle + \frac{1}{2t} \Vert v \Vert ^2- \frac{1}{t} \langle v,u_{n_k}\rangle =:H(v)\Big \rbrace . \end{aligned}$$

Clearly, $\partial H(v)=\partial h(v) + \nabla j(u_{n_k}) + \tfrac{1}{t} (v-u_{n_k})$. By optimality of $\bar{u}_{n_k+1}$ (see Fermat’s rule, [4, Theorem 16.2]), $0 \in \partial H(\bar{u}_{n_k+1})$, or equivalently,

$$\begin{aligned} -\frac{1}{t} (\bar{u}_{n_k+1} - u_{n_k}) \in \nabla j(u_{n_k}) + \partial h(\bar{u}_{n_k+1}). \end{aligned}$$

Taking the limit as $k \rightarrow \infty$, and using continuity of $\nabla j$, we conclude by strong-to-weak sequential closedness of $\mathrm{gra} (\partial h)$ that

3.15 $$\begin{aligned} 0 \in \nabla j(u) + \partial h(u), \end{aligned}$$

so therefore *u* is a stationary point. $\square$

### Stochastic proximal gradient method: decreasing step sizes

An obvious drawback of Algorithm 1 is the fact that step sizes are restricted to small steps bounded by a factor depending on the Lipschitz constant, which in applications might be difficult to determine. Additionally, the algorithm requires increasing batch sizes to dampen noise, which is unattractive from a complexity standpoint. In this section, we obtain convergence with a nonsmooth and convex term *h* using the step size rule

3.16 $$\begin{aligned} t_n \ge 0, \quad \sum _{n=1}^\infty t_n = \infty , \quad \sum _{n=1}^\infty t_n^2 < \infty . \end{aligned}$$

This step size rule dampens noise enough so that increased sampling is not necessary.

We observe Problem (P) with

$$\begin{aligned} h(u) := \eta (u) + \delta _C(u). \end{aligned}$$

For asymptotic arguments, it will be convenient to treat the term $\delta _C$ separately. To that end, we define

$$\begin{aligned} \varphi (u):=j(u)+\eta (u) \end{aligned}$$

and note that $f(u) = \varphi (u) + \delta _C(u).$ The stochastic gradient $G(u,\xi ):H\times \varXi \rightarrow H$ can be comprised of one or more samples as in the unconstrained case; see Remark 3.2. The algorithm is now stated below. 
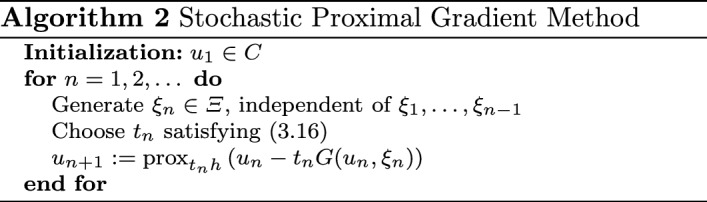


To prove convergence of Algorithm 2, we will use the ODE method, which dates back to [33, 36]. While we use many ideas from [13], we emphasize that we generalize results to (possibly infinite-dimensional) Hilbert spaces and moreover, we handle the case when *j* is the expectation.

We define the set-valued map $S:C \rightrightarrows H$ by

$$\begin{aligned} S(u) := - \nabla j(u) - \partial \eta (u) - N_C(u). \end{aligned}$$

Additionally, we define the sequence of (single-valued) maps $S_n:C \rightarrow H$ for all *n* by

$$\begin{aligned} S_n (u):= - \nabla j(u) - \frac{1}{t_n} \mathbb {E}[u - t_n G(u,\xi ) - \text {prox}_{t_n h}(u - t_n G(u,\xi ))]. \end{aligned}$$

In addition to Assumption 3.1, the following assumptions will apply in this section.

#### Assumption 3.11

Let $\{ u_n\}$ and $\{G(u_n,\xi _n)\}$ be generated by Algorithm 2. We assume

(i) The set *C* is nonempty, bounded, convex, and closed.
(ii) The function $\eta \in \varGamma _0(H)$ with $\mathrm{dom}(\eta ) = H$ is locally Lipschitz and bounded below on *C*, and there exists a function $L_{\eta }: H \rightarrow \mathbb {R}$, which is bounded on bounded sets, satisfying
  3.17 $$\begin{aligned} L_\eta (u) \ge \sup _{z:\eta (z) \le \eta (u)} \frac{\eta (u)-\eta (z)}{\Vert u - z\Vert }. \end{aligned}$$
(iii) There exists a function $M:H \rightarrow [0,\infty )$, which is bounded on bounded sets, such that $\mathbb {E}[\Vert G(u,\xi ) \Vert ^2] \le M(u).$
(iv) For any strongly convergent sequence $\{u_n\}$, $\mathbb {E}[ \sup _n \Vert G(u_n,\xi ) \Vert ] < \infty$ holds.
(v) The set of critical values $\{ f(u): 0 \in \partial f(u)\}$ does not contain any segment of nonzero length.

#### Remark 3.12

To handle the infinite-dimensional case, we use assumptions that are generally more restrictive than in [13]; we restrict ourselves to the case where *C* and $\eta$ are convex and we assume higher regularity of *j* in Assumption 3.1(ii) to handle the case $j(u) = \mathbb {E}[J(u,\xi )]$. However, we allow for bias $r_n$, which is not covered in [13]. We note that *C* does not need to be bounded if $\eta$ is Lipschitz continuous over *C*. Assumption 3.11(ii) is satisfied if $\mathrm{dom}(\partial \eta ) = H$ and $\partial \eta$ maps bounded sets to bounded sets; see also [4, Proposition 16.17] for equivalent conditions. The last assumption is technical but standard; see [48, Assumption H4].

The main result is the following, which we will prove in several parts. Throughout, we use the notation $g_n:=G(u_n,\xi _n)$.

#### Theorem 3.13

*Let Assumptions* 3.1*and* 3.11*hold. Then*

1. *The sequence* $\{ f(u_n) \}$ *converges a.s.*
2. *Every strong accumulation point* *u* *of the sequence* $\{u_n\}$ *is a stationary point with probability one, namely,* $0 \in \partial f(u)$ *a.s.*

#### Lemma 3.14

*The sequence* $\{ u_n\}$ *satisfies the recursion*

3.18 $$\begin{aligned} u_{n+1} = u_n + t_n (y_n - r_n +w_n), \end{aligned}$$

*where* $$y_n = S_n(u_n)$$ *and* $$w_n=-\frac{1}{t_n}\mathbb {E}[\text {prox}_{t_n h}(u_n - t_n g_n)|\mathcal {F}_n] + \frac{1}{t_n}\text {prox}_{t_n h}(u_n - t_n g_n)$$.

#### Proof

Note that $u_n$ and $r_n$ are $\mathcal {F}_n$-measurable, so $\mathbb {E}[g_n|\mathcal {F}_n] = \nabla j(u_n) + r_n$. Then

$$\begin{aligned}&u_{n+1} - u_n =\text {prox}_{t_n h}(u_n - t_n g_n) - u_n\\&\quad = -t_n \mathbb {E}[g_n |\mathcal {F}_n] - \mathbb {E}[u_n - t_n g_n - \text {prox}_{t_n h}(u_n - t_n g_n)|\mathcal {F}_n] \\&\qquad - \mathbb {E}[\text {prox}_{t_n h}(u_n - t_n g_n)|\mathcal {F}_n] + \text {prox}_{t_n h}(u_n - t_n g_n)\\&\quad = t_n S_n(u_n) - t_n r_n - \mathbb {E}[\text {prox}_{t_n h}(u_n - t_n g_n)|\mathcal {F}_n] + \text {prox}_{t_n h}(u_n - t_n g_n), \end{aligned}$$

where we used that $\xi _n$ is independent from $\xi _1, \dots , \xi _{n-1}$, so

3.19 $$\begin{aligned} \begin{aligned}&\mathbb {E}[u_n - t_n g_n - \text {prox}_{t_n h}(u_n - t_n g_n)|\mathcal {F}_n]\\&\quad = \mathbb {E}[u_n - t_n G(u_n,\xi ) - \text {prox}_{t_n h}(u_n - t_n G(u_n,\xi ))]. \end{aligned} \end{aligned}$$

By definition of $y_n$ and $w_n$, we arrive at the conclusion. $\square$

#### Lemma 3.15

*For any* $u \in C$, $g \in H$ *and* $t > 0$, *we have for* $\bar{u} = \text {prox}_{t h}(u - t g)$ *that*

$$\begin{aligned} \frac{1}{t} \Vert \bar{u} - u \Vert \le 2 L_\eta (u) + 2 \Vert g \Vert . \end{aligned}$$

#### Proof

By definition of the proximity operator,

$$\begin{aligned} \eta (\bar{u}) + \delta _C(\bar{u})+\frac{1}{2t} \Vert \bar{u} - (u-tg) \Vert ^2 \le \eta (u)+ \delta _C({u}) +\frac{1}{2t} \Vert u - (u-tg) \Vert ^2, \end{aligned}$$

or equivalently (note $\bar{u}, u \in C$),

$$\begin{aligned} \eta (\bar{u}) + \frac{1}{2t} \Vert \bar{u} -u \Vert ^2 + \langle \bar{u}-u, g\rangle \le \eta (u). \end{aligned}$$

By (3.17), in the case $\eta (u) \ge \eta (\bar{u})$, we obtain

3.20 $$\begin{aligned} \frac{1}{t} \Vert \bar{u} - u \Vert ^2 \le 2 (\eta (u) - \eta (\bar{u})) - 2\langle \bar{u} - u, g\rangle \le 2L_\eta (u) \Vert \bar{u} - u \Vert + 2\Vert \bar{u} - u \Vert \Vert g \Vert . \end{aligned}$$

Notice that the last inequality (3.20) is trivial whenever $\eta (u) \le \eta (\bar{u})$. This yields the conclusion. $\square$

#### Lemma 3.16

*The sequence* $\{ y_n\}$ *is bounded a.s.*

#### Proof

By the characterization of $y_n=S_n(u_n)$ from Lemma 3.14 and (3.19), followed by Jensen’s inequality, and the application of Lemma 3.15 in the fourth inequality, we get

3.21 $$\begin{aligned} \begin{aligned} \Vert y_n \Vert&\le \Vert \nabla j(u_n)\Vert + \Vert \tfrac{1}{t_n}\mathbb {E}[u_n - t_n g_n - \text {prox}_{t_n h}(u_n - t_n g_n)|\mathcal {F}_n] \Vert \\&\le \Vert \nabla j(u_n)\Vert + \mathbb {E}\big [\Vert \tfrac{1}{t_n}\big (u_n - t_n g_n - \text {prox}_{t_n h}(u_n - t_n g_n)\big ) \Vert |\mathcal {F}_n\big ]\\&\le \Vert \nabla j(u_n)\Vert + \mathbb {E}[\Vert g_n\Vert |\mathcal {F}_n]+ \mathbb {E}\big [ \Vert \tfrac{1}{t_n}\big (u_n - \text {prox}_{t_n h}(u_n - t_n g_n)\big ) \Vert |\mathcal {F}_n\big ]\\&\le \Vert \nabla j(u_n)\Vert + \mathbb {E}[\Vert g_n\Vert |\mathcal {F}_n]+ 2 L_{\eta }(u_n) + 2 \mathbb {E}[\Vert g_n \Vert |\mathcal {F}_n]\\&\le \Vert \nabla j(u_n)\Vert + 3\sqrt{M(u_n)} + 2 L_{\eta }(u_n). \end{aligned} \end{aligned}$$

The last step follows by $\mathbb {E}[\Vert g_n\Vert | \mathcal {F}_n] = \mathbb {E}[\Vert G(u_n,\xi )\Vert ]$ and Assumption 3.11(iii) with Jensen’s inequality. We have from Assumption 3.1(i) that $\{ u_n\}$ is bounded a.s.; therefore, all terms on the right-hand side of (3.21) are bounded a.s. $\square$

For Lemma 3.18, we need the following result, which is a generalization of a convergence theorem for quadratic variations from [57, p. 111] to Bochner spaces. The proof can be found in Sect. A.

#### Lemma 3.17

*Let* $\{v_n\}$ *be an* *H*-*valued martingale. Then* $\{v_n\}$ *is bounded in* $L^2(\varOmega ,H)$ *if and only if*

3.22 $$\begin{aligned} \sum _{n=1}^\infty \mathbb {E}[\Vert v_{n+1}-v_n\Vert ^2] < \infty , \end{aligned}$$

*and when this is satisfied,* $v_n \rightarrow v_\infty$ *a.s. as* $n \rightarrow \infty$.

#### Lemma 3.18

*The series* $\sum _{j=1}^N t_j w_j$ *a.s. converges to a limit as* $N \rightarrow \infty$.

#### Proof

Recall the elementary inequality $\mathbb {E}[\Vert X - \mathbb {E}[X|\mathcal {F}_n]\Vert ^2|\mathcal {F}_n]\le \mathbb {E}[\Vert X\Vert ^2|\mathcal {F}_n]$, which holds for any random variable *X*. By Lemma 3.14 with

$$\begin{aligned} X:=\tfrac{1}{t_n}(\text {prox}_{t_n h}(u_n - t_n g_n)-u_n), \end{aligned}$$

followed by Lemma 3.15 and Assumption 3.11(iii), we get

3.23 $$\begin{aligned} \begin{aligned} \mathbb {E}[\Vert w_n \Vert ^2 | \mathcal {F}_n]&\le \tfrac{1}{t_n^2}\mathbb {E}[\Vert \text {prox}_{t_n h}(u_n - t_n g_n) - u_n\Vert ^2 | \mathcal {F}_n] \\&\le 4 (L_{\eta }(u_n))^2 +4M(u_n) <\infty . \end{aligned} \end{aligned}$$

Let $v_n := \sum _{j=1}^n t_j w_j$. We show that $v_n$ is a square integrable martingale, i.e., $v_n \in L^2(\varOmega , H)$ for every *n* and $\sup _{n} \mathbb {E}[\Vert v_n \Vert ^2]<\infty .$ It is clearly a martingale, since for all *n*, $\mathbb {E}[w_n|\mathcal {F}_n] = 0$ and thus

$$\begin{aligned} \mathbb {E}[v_n|\mathcal {F}_n] = \mathbb {E}[t_n w_n |\mathcal {F}_n] + \sum _{j=1}^{n-1} t_j w_j = v_{n-1}. \end{aligned}$$

To show that $v_n$ is square integrable, we use (3.23) and the fact that $\mathbb {E}[v_n]=0$ for all *n* to conclude that its quadratic variations are bounded. Indeed,

$$\begin{aligned} A_n&:= \sum _{j=2}^n \mathbb {E}[\Vert v_{j} - v_{j-1} \Vert ^2 | \mathcal {F}_{j}]= \sum _{j=2}^{n} t_j^2 \mathbb {E}[\Vert w_j \Vert ^2|\mathcal {F}_j]. \end{aligned}$$

Because of the condition (3.16), we have that $\sup _n \mathbb {E}[A_n] < \infty .$ We have obtained that $\{v_n\}$ is square integrable, so by Lemma 3.17, it follows that $\{v_n\}$ converges a.s. to a limit as $n\rightarrow \infty$. $\square$

#### Lemma 3.19

*The following is true with probability one:*

3.24 $$\begin{aligned} \lim _{n \rightarrow \infty } \Vert u_{n+1} - u_n \Vert = 0. \end{aligned}$$

#### Proof

This is a simple consequence of (3.18) and a.s. boundedness of $y_n$, $r_n$, and $w_n$ for all *n* by Lemmas 3.16, 3.18, and Assumption 3.1(iii), respectively. $\square$

#### Lemma 3.20

*For any sequence* $\{z_n\}$ *in* *C* *such that* $z_n \rightarrow z$ *as* $n \rightarrow \infty$*, it follows that*

3.25 $$\begin{aligned} \lim _{m\rightarrow \infty } d\left( \frac{1}{m} \sum _{n=1}^{m} S_{n}(z_{n}), S(z) \right) = 0 \quad \text {a.s.} \end{aligned}$$

#### Proof

Notice that *C* is closed, so $z\in C$. The fact that *S*(*z*) is nonempty, closed, and convex follows by these properties of $\nabla j(z)$, $\partial \eta (z)$, and $N_C(z)$. We define $g_n^\xi :=G(z_n,\xi )$ and

3.26 $$\begin{aligned} \tilde{S}_n(z_n, \xi ):=- \nabla j(z_n) - \frac{1}{t_n} (z_n -t_n g_n^\xi - \text {prox}_{t_n h}(z_n - t_n g_n^\xi )). \end{aligned}$$

Clearly, $\mathbb {E}_{\xi }[\tilde{S}_n(z_n, \xi )] = S_n(z_n).$ Now, by Jensen’s inequality and convexity of the mapping $u \mapsto d (u,S(z))$,

$$\begin{aligned} d\left( \frac{1}{m} \sum _{n=1}^{m} S_{n}(z_{n}),S(z) \right)&\le \frac{1}{m} \sum _{n=1}^{m} d(S_n(z_n),S(z))\\&\le \frac{1}{m} \sum _{n=1}^{m} \mathbb {E}_{\xi }\left[ d(\tilde{S}_n(z_n, \xi ),S(z))\right] . \end{aligned}$$

Notice that $\bar{z} = \text {prox}_{t h}(u)$ if and only if $0 \in \partial \eta (\bar{z})+ N_C(\bar{z}) +\tfrac{1}{t}(\bar{z}-u)$, so with

3.27 $$\begin{aligned} \bar{z}_n:=\text {prox}_{t_n h}(z_n -t_n g_n^\xi ), \end{aligned}$$

there exist $\zeta _{\eta ,n} \in \partial \eta (\bar{z}_n)$ and $\zeta _{C,n} \in N_C(\bar{z}_n)$ such that

3.28 $$\begin{aligned} -(\zeta _{\eta ,n} + \zeta _{C,n}) = \frac{1}{t_n}(\bar{z}_n - z_n + t_n g_n^\xi ). \end{aligned}$$

Because $\{ z_n\}$ converges, it is contained in a bounded set. Hence, by Lemma 3.15, we get

3.29 $$\begin{aligned} \begin{aligned} \Vert \zeta _{\eta ,n} + \zeta _{C,n} \Vert& = \tfrac{1}{t_n} \Vert \bar{z}_n - z_n + t_n g_n^\xi \Vert \le 2 L_{\eta }(z_n) + 3\Vert g_n^\xi \Vert , \end{aligned} \end{aligned}$$

which must be almost surely finite by Assumption 3.11(iv). Now, by (3.26) and (3.27), followed by (3.28),

$$\begin{aligned} d(\tilde{S}_n(z_n, \xi ),S(z))& = d(- \nabla j(z_n) + \tfrac{1}{t_n} (\bar{z}_n - z_n +t_n g_n^\xi ),S(z))\\& = d(- \nabla j(z_n) - \zeta _{\eta ,n} - \zeta _{C,n}, S(z)). \end{aligned}$$

By the simple rule $d(u+v,A+B) \le d(u,A)+d(v,B)$ for sets *A* and *B* and points $u, v\in H$, we get by definition of *S*(*z*) that

$$\begin{aligned} d(\tilde{S}_n(z_n, \xi ),S(z)) \le \Vert \nabla j(z_n)-\nabla j(z)\Vert + d(\zeta _{\eta ,n},\partial \eta (z)) + d(\zeta _{C,n},N_C(z)). \end{aligned}$$

By strong-to-weak sequential closedness of $\mathrm{gra} (\partial \eta )$ and $\mathrm{gra} ( N_C)$ as well as continuity of $\nabla j$, it follows that

3.30 $$\begin{aligned} \lim _{n \rightarrow \infty } d(\tilde{S}_n(z_n, \xi ),S(z)) = 0 \quad \text {a.s.} \end{aligned}$$

We show that $d(\tilde{S}_n(z_n, \xi ),S(z))$ is almost surely bounded by an integrable function $\tilde{M}(z)$ for all *n*. Using elementary arguments and (3.29) in the third inequality,

$$\begin{aligned}&d(\tilde{S}_n(z_n, \xi ),S(z))\\&\quad \le \quad d(-\nabla j(z_n) -\zeta _{\eta ,n} - \zeta _{C,n},S(z))\\&\quad \le \quad \Vert \nabla j(z_n) -\nabla j(z)\Vert + d (\zeta _{\eta ,n} + \zeta _{C,n},\partial \eta (z) + N_C(z))\\&\quad \le \quad \Vert \nabla j(z_n) - \nabla j(z)\Vert + 2 L_{\eta }(z_n) + 3\Vert g_n^\xi \Vert + d(0,\partial \eta (z) + N_C(z))\\&\quad \le \quad \sup _{n \in \mathbb {N}} \left\{ \Vert \nabla j(z_n) - \nabla j(z)\Vert + 2 L_{\eta }(z_n) + 3\Vert g_n^\xi \Vert + d(0,\partial \eta (z) + N_C(z))\right\} , \end{aligned}$$

which is almost surely bounded by Assumption 3.11(ii) and Assumption 3.11(iv). By the dominated convergence theorem, it follows by (3.30) that as $n \rightarrow \infty$, $\mathbb {E}_{\xi }[d(\tilde{S}_n(z_n, \xi ),S(z))]\rightarrow 0$. Finally, (3.25) follows from the fact that if $a_n \rightarrow 0$ as $n \rightarrow \infty$, it follows that $\tfrac{1}{m}\sum _{n=1}^m a_n \rightarrow 0$ as $m \rightarrow \infty$. $\square$

Now we will show a compactness result, adapted from [15], namely that in the limit, the time shifts of the linear interpolation of the sequence $\{ u_n\}$ can be made arbitrarily close to trajectories, or solutions, of the differential inclusion

3.31 $$\begin{aligned} \dot{z}(t) \in S(z(t)). \end{aligned}$$

The set *C*(*I*, *H*) denotes the space of continuous functions from *I* to *H*. We recall that if $z(\cdot ) \in C([0,\infty ),H)$ satisfies (3.31) and is absolutely continuous on any compact interval $[a,b] \subset (0,\infty )$, it is called a strong solution. The existence and uniqueness of this solution is guaranteed by the following result.

#### Proposition 3.21

*For every* $z_0=z(0)\in C$ *there exists a unique strong solution* $z \in C([0,\infty ),H)$ *to the differential inclusion* (3.31).

#### Proof

The function $u \mapsto \eta (u) + \delta _C(u)$ is proper, convex, and lower semicontinuous and $B := - \nabla j$ is Lipschitz continuous. Therefore, by [8, Proposition 3.12], the statement follows. $\square$

For the next result, we set $s_n: = \sum _{j=1}^{n-1} t_j$ and define the linear interpolation $u:[0,\infty ) \rightarrow H$ of iterates as well as the piecewise constant extension $y:[0,\infty ) \rightarrow H$ of the sequence $\{y_n\}$ via

3.32 $$\begin{aligned} u(t) := u_n + \frac{t-s_n}{s_{n+1}-s_n} (u_{n+1} - u_n), \quad y(t) := y_n, \quad \forall t \in [s_n,s_{n+1}), \forall n \in \mathbb {N}. \end{aligned}$$

The time shifts of $u(\cdot )$ are denoted by $u(\cdot +\tau )$ for $\tau >0$. We define $u^\tau :[0,\infty ) \rightarrow H$ by

3.33 $$\begin{aligned} u^\tau (t):=u(\tau ) + \int _\tau ^{t} y(s) \,\mathrm {d}s \end{aligned}$$

as the solution to the ODE

$$\begin{aligned} \dot{u}^\tau (\cdot ) = y(\cdot ), \quad u^\tau (\tau ) = u(\tau ), \end{aligned}$$

which is guaranteed to exist by [9, Theorem 1.4.35].

#### Theorem 3.22

*For any* $T>0$ *and any nonnegative sequence* $\{ \tau _n\}$, *the sequence of the time shifts* $\{ u(\cdot +\tau _n)\}$ *is relatively compact in* *C*([0, *T*], *H*). *If* $\tau _n \rightarrow \infty$, *all limit points* $\bar{u}(\cdot )$ *of the time shifts* $\{ u(\cdot +\tau _n)\}$ *are in* *C*([0, *T*], *H*) *and there exists a* $\bar{y}:[0,T] \rightarrow H$ *such that* $\bar{y}(t) \in S(\bar{u}(t))$ *and* $\bar{u}(t) = \bar{u}(0) + \int _0^t \bar{y}(s) \,\mathrm {d}s.$

#### Proof

**Relative compactness of time shifts.** We first claim that for all $T>0$,

3.34 $$\begin{aligned} \lim _{\tau \rightarrow \infty } \sup _{t \in [\tau , \tau +T]} \Vert u^{\tau }(t)-u(t) \Vert = 0 \quad \text {a.s.} \end{aligned}$$

We consider a fixed (but arbitrary) sample path $\omega = (\omega _1, \omega _2, \dots )$ throughout the proof. Let $p:=\min \{n:s_n \ge \tau \}$ and $q:=\max \{n:s_n \le t\}$. By (3.33) and (3.32),

3.35 $$\begin{aligned} \begin{aligned} u^\tau (t)& = u(\tau ) + \int _{\tau }^t y(s) \,\mathrm {d}s = u(\tau ) + \int _{\tau }^{s_{p}} y(s) \,\mathrm {d}s + \sum _{\ell =p}^{q-1} t_\ell y_\ell + \int _{s_{q}}^t y(s) \,\mathrm {d}s. \end{aligned} \end{aligned}$$

Notice that due to the recursion (3.18),

3.36 $$\begin{aligned} \sum _{\ell = p}^{q-1} t_\ell y_\ell = u_{q} - u_{p} - \sum _{\ell =p}^{q-1} t_\ell (w_\ell -r_\ell ). \end{aligned}$$

Plugging (3.36) into (3.35), we get

$$\begin{aligned} u^\tau (t) - u(t)& = u(\tau ) + u_{q} - u_{p} - u(t) + \int _{\tau }^{s_{p}} y(s) \,\mathrm {d}s \\&\quad - \sum _{\ell =p}^{q-1} t_\ell (w_\ell - r_\ell )+ \int _{s_{q}}^t y(s) \,\mathrm {d}s. \end{aligned}$$

Therefore,

$$\begin{aligned} \Vert u^\tau (t) - u(t) \Vert&\le \left\Vert u(\tau ) - u_{p} +\int _{\tau }^{s_{p}}y(s) \,\mathrm {d}s \right\Vert + \left\Vert u_{q} - u(t) +\int _{s_{q}}^{t} y(s) \,\mathrm {d}s\right\Vert \\&\quad + \left\Vert \sum _{\ell =p}^{q-1} t_\ell w_\ell \right\Vert + \left\Vert \sum _{\ell =p}^{q-1} t_\ell r_\ell \right\Vert . \end{aligned}$$

Note that by (3.32), it follows that

$$\begin{aligned} \Vert u(\tau ) - u_{p} \Vert&\le \Vert u_{p-1} - u_{p}\Vert = t_{p-1} \Vert y_{p-1} - r_{p-1} + w_{p-1}\Vert , \\ \Vert u_{q} - u(t)\Vert&\le \Vert u_{q} - u_{q+1}\Vert = t_{q}\Vert y_{q} - r_{q} + w_{q}\Vert . \end{aligned}$$

Moreover, by (3.32), we have

$$\begin{aligned} \left\Vert \int _{\tau }^{s_{p}} y(s) \,\mathrm {d}s\right\Vert \le t_{p-1} \Vert y_{p-1}\Vert \quad \text {and} \quad \left\Vert \int _{s_{q}}^{t} y(s) \,\mathrm {d}s\right\Vert \le t_{q} \Vert y_{q}\Vert . \end{aligned}$$

Therefore,

3.37 $$\begin{aligned} \begin{aligned} \Vert u^\tau (t) - u(t) \Vert&\le t_{p-1} (2\Vert y_{p-1}\Vert +\Vert r_{p-1}\Vert + \Vert w_{p-1}\Vert )\\&\quad +t_{q} (2\Vert y_{q}\Vert +\Vert r_{q}\Vert + \Vert w_{q}\Vert ) + \left\Vert \sum _{\ell =p}^{q-1} t_\ell w_\ell \right\Vert + \left\Vert \sum _{\ell =p}^{q-1} t_\ell r_\ell \right\Vert . \end{aligned} \end{aligned}$$

We take the limit $p,q \rightarrow \infty$ on the right-hand side of (3.37) and observe that by Lemma 3.16, $\lim _{n \rightarrow \infty } \sup _{m \ge n} t_m \Vert y_m \Vert = 0$ and by Lemma 3.18, we have $\lim _{n \rightarrow \infty } \sup _{m \ge n} \Vert \sum _{\ell =n}^{m-1} t_\ell w_\ell \Vert = 0$ as well as $\lim _{n \rightarrow \infty } \sup _{m\ge n} t_m \Vert w_m\Vert$. By Assumption 3.1(iii), we have $\lim _{n \rightarrow \infty } \sup _{m \ge n} \left\Vert \sum _{\ell =n}^{m-1} t_\ell r_\ell \right\Vert = 0.$ We have shown (3.34), so it follows that the set

$$\begin{aligned} A:=\{u^\tau (\cdot ): \tau \in [0,\infty )\} \end{aligned}$$

is a family of equicontinuous functions.

To invoke the Arzelà–Ascoli theorem, we first show that the set

$$\begin{aligned} A(t):=\{ u^{\tau }(t): \tau \in [0,\infty )\} \end{aligned}$$

is relatively compact for all $t\in [0,T]$, $T>0$. We show this by proving that arbitrary sequences in *A*(*t*) have a Cauchy subsequence, which converge in *H* by completeness of *H*. To this end, let $\varepsilon >0$ be arbitrary and observe first the case $\tau _n \rightarrow \infty .$ Let $n_k$ be the index such that $\tau _k \in [s_{n_k}, s_{n_k+1})$ and

$$\begin{aligned} u^{\tau _k}(t) = u_{n_k} + \frac{\tau _k - s_{n_k}}{s_{n_k+1}-s_{n_k}} (u_{n_k+1} - u_{n_k}) + \int _{\tau _k}^t y(s) \,\mathrm {d}s. \end{aligned}$$

Similarly, let $m_j$ be the index such that $\tau _j \in [s_{m_j},s_{m_j+1})$. Thus we have

3.38 $$\begin{aligned} \begin{aligned}&\Vert u^{\tau _k}(t) - u^{\tau _j}(t)\Vert \\&\quad \le \left\Vert \frac{\tau _k - s_{n_k}}{s_{n_k+1}-s_{n_k}} ( u_{n_k+1} - u_{n_k}) - \frac{\tau _j - s_{m_j}}{s_{m_j+1}-s_{m_j}} ( u_{m_j+1} - u_{m_j}) \right\Vert \\&\qquad + \left\Vert u_{n_k} - u_{m_j} + \int _{\tau _k}^{\tau _j} y(s) \,\mathrm {d}s\right\Vert . \end{aligned} \end{aligned}$$

Using (3.36), we get (w.l.o.g. $\tau _k \le \tau _j$)

3.39 $$\begin{aligned} \begin{aligned} \left\Vert u_{n_k} - u_{m_j} + \int _{\tau _k}^{\tau _j} y(s) \,\mathrm {d}s\right\Vert&\le \Vert u_{n_k} - u_{n_k+1} \Vert + \left\Vert \int _{\tau _k}^{s_{n_k+1}} y(s) \,\mathrm {d}s \right\Vert \\&\quad +\left\Vert \int _{s_{m_j}}^{\tau _j} y(s) \,\mathrm {d}s \right\Vert +\left\Vert \sum _{\ell =n_k+1}^{m_j-1} t_\ell (w_\ell - r_\ell )\right\Vert . \end{aligned} \end{aligned}$$

Combining (3.38) and (3.39), and observing that $\left| \tfrac{\tau _k - s_{n_k}}{s_{n_k+1}-s_{n_k}}\right| \le 1$ as well as $\left| \tfrac{\tau _j - s_{m_j}}{s_{m_j+1}-s_{m_j}}\right| \le 1$, we obtain

3.40 $$\begin{aligned} \begin{aligned} \Vert u^{\tau _k}(t) - u^{\tau _j}(t)\Vert&\le 2 \Vert u_{n_k+1} - u_{n_k}\Vert + \Vert u_{m_j+1} - u_{m_j}\Vert + t_{n_k} \Vert y_{n_k}\Vert \\&\quad + t_{m_j} \Vert y_{m_j}\Vert + \left\Vert \sum _{\ell =n_k+1}^{m_j-1} t_\ell (w_\ell - r_\ell )\right\Vert . \end{aligned} \end{aligned}$$

By Lemma 3.19 as well as convergence of the other terms on the right-hand side of (3.40), for $\varepsilon >0$ there exists a *N* such that for all $k, j > N$, $\Vert u^{\tau _k}(t) - u^{\tau _j}(t)\Vert \le \varepsilon$ for all $k, j > N$ and thus $\{ u^{\tau _n}(t)\}$ has a Cauchy subsequence for $\tau _n \rightarrow \infty$. Now we observe the case where the sequence $\{\tau _n \}$ is bounded. Then $\tau _n \rightarrow \bar{\tau }$ for some $\bar{\tau }>0$ at least on a subsequence (with the same labeling). By convergence of $\{\tau _n\}$ we get that $m_j=n_k$ for $k, j \ge N$ and *N* large enough. Therefore (3.38) reduces to

3.41 $$\begin{aligned} \Vert u^{\tau _k}(t) - u^{\tau _j}(t)\Vert \le \left|\frac{\tau _k - \tau _j}{s_{n_k+1}-s_{n_k}}\right|\Vert u_{n_k+1}-u_{n_k} \Vert + \left\Vert \int _{\tau _k}^{\tau _j} y(s) \,\mathrm {d}s \right\Vert . \end{aligned}$$

We can bound terms on the right-hand side of (3.41) as before to obtain that $\{ u^{\tau _n}(t) \}$ has a Cauchy subsequence. We have shown that *A*(*t*) is relatively compact for all $t\in [0,T]$, $T>0$, so by the Arzelà–Ascoli theorem, it follows that the set *A* is relatively compact.

Now, the relative compactness of the set of time shifts $\{ u(\cdot +\tau ): \tau \in [0,\infty )\}$ follows from the relative compactness of the set *A*. Indeed, for any sequence $\{u^{\tau _n}(\cdot + \tau _n)\}$ there exists a convergent subsequence such that $u^{\tau _{n_k}}(\cdot + \tau _{n_k}) \rightarrow \bar{u}(\cdot )$ for some $\bar{u}(\cdot ) \in C([0,T],H)$. Now, for the time shift $u(\cdot +\tau _{n_k})$, we have

$$\begin{aligned}&\sup _{t \in [0,T]}\Vert u(t+\tau _{n_k}) - \bar{u}(t)\Vert \\&\quad \le \sup _{t \in [0,T]}\Vert u(t+\tau _{n_k}) - u^{\tau _{n_k}}(t+\tau _{n_k})\Vert + \sup _{t \in [0,T]}\Vert u^{\tau _{n_k}}(t+\tau _{n_k}) - \bar{u}(t)\Vert , \end{aligned}$$

so it follows that $u(\cdot +\tau _{n_k}) \rightarrow \bar{u}(\cdot )$ in *C*([0, *T*], *H*) as $\tau _{n_k}\rightarrow \infty$ by convergence of $u^{\tau _{n_k}}(\cdot )$ and (3.34). If $\tau _{n_k} \rightarrow \bar{\tau }$, then $u(\cdot +\tau _{n_k}) \rightarrow u(\cdot +\bar{\tau })$ by uniform continuity of $u(\cdot )$ on $[0,\bar{\tau }+T].$

*Limit points are trajectories of the differential inclusion*. Let $\{ \tau _n\}$ be a sequence such that as $\tau _n \rightarrow \infty$, $u^{\tau _n}(\cdot +\tau _n) \rightarrow \bar{u}(\cdot )$ in *C*([0, *T*], *H*) (potentially on a subsequence). The sequence $\{ y(\cdot +\tau _n) \} \subset L^2([0,T], H)$ is bounded by boundedness of $\{ y_n\}$, and since $L^2([0,T], H)$ is a Hilbert space, there exists a subsequence $\{ n_k\}$ such that $y(\cdot +\tau _{n_k}) \rightharpoonup \bar{y}(\cdot )$ in $L^2([0,T],H)$ for some $\bar{y} \in L^2([0,T],H)$. Notice that for $\{\tau _{n_k}\}$, by (3.33) it follows that

3.42 $$\begin{aligned} u^{\tau _{n_k}}(t+\tau _{n_k}) = u^{\tau _{n_k}}(\tau _{n_k}) + \int _0^t y(s+\tau _{n_k}) \,\mathrm {d}s. \end{aligned}$$

By (3.34), $u^{\tau _{n_k}}( \cdot +\tau _{n_k}) \rightarrow \bar{u}(\cdot )$ in *C*([0, *T*], *H*) as $k \rightarrow \infty$. Taking $k \rightarrow \infty$ on both sides of (3.42) we get, due to $y(\cdot +\tau _{n_k}) \rightharpoonup \bar{y}(\cdot )$ for $t \in [0,T]$, that

$$\begin{aligned} \bar{u}(t) = \bar{u}(0) + \int _0^t \bar{y}(s) \,\mathrm {d}s. \end{aligned}$$

Now, we will show that $\bar{y}(t) \in S(\bar{u}(t))$ for a.e. $t \in [0,T]$. By the Banach-Saks theorem (cf. [42]), there exists a subsequence of $\{ y(\cdot +\tau _{n_k})\}$ (where we use the same notation for the sequence as its subsequence) such that

3.43 $$\begin{aligned} \lim _{m \rightarrow \infty }\frac{1}{m} \sum _{k=1}^m y(\cdot +\tau _{n_k}) = \bar{y}(\cdot ). \end{aligned}$$

Recall that $y_n = S_n(u_n)$ by Lemma 3.14 and set $\ell _k^t:= \max \{ \ell : s_\ell \le t+\tau _{n_k}\}.$ Then we have

$$\begin{aligned} y(t+\tau _{n_k}) = y(s_{\ell _k^t}) = y_{\ell _k^t} = S_{\ell _k^t}(u_{\ell _k^t}). \end{aligned}$$

Therefore, since $$t+\tau _{n_k} \in [\ell _k^t, \ell _k^t+1]$$,

3.44 $$\begin{aligned} \begin{aligned} \Vert u(s_{\ell _k^t}) - \bar{u}(t)\Vert&\le \Vert u(s_{\ell _k^t}) - u(t+\tau _{n_k}) \Vert + \Vert u(t+\tau _{n_k}) - \bar{u}(t) \Vert \\&\le \Vert u(s_{\ell _k^t}) - u(s_{\ell _k^t+1}) \Vert + \Vert u(t+\tau _{n_k}) - \bar{u}(t) \Vert \\&\le t_{\ell _k^t} (\Vert y_{\ell _k^t}\Vert + \Vert r_{\ell _k^t}\Vert + \Vert w_{\ell _k^t}\Vert ) +\Vert u(t+\tau _{n_k}) - \bar{u}(t) \Vert , \end{aligned} \end{aligned}$$

which a.s. converges to zero as $k \rightarrow \infty$, since $u(\cdot +\tau _{n_k}) \rightarrow \bar{u}(\cdot )$ and the fact that $t_{n} \rightarrow 0$ by (3.16) (combined a.s. boundedness of $y_n, r_n$, and $w_n$ for all *n* by Lemma 3.16, Assumption 3.1(iii), and Lemma 3.18, respectively). Now, using $y(t+\tau _{n_k}) = y_{\ell _k^t}$, we get

$$\begin{aligned}&d(\bar{y}(t),S(\bar{u}(t)))\\&\quad \le \left\Vert \frac{1}{m} \sum _{k=1}^m y(t+\tau _{n_k}) - \bar{y}(t) \right\Vert + d\left( \frac{1}{m} \sum _{k=1}^m y(t+\tau _{n_k}), S(\bar{u}(t))\right) \\&\quad \le \left\Vert \frac{1}{m} \sum _{k=1}^m y(t+\tau _{n_k}) - \bar{y}(t) \right\Vert + d\left( \frac{1}{m} \sum _{k=1}^m S_{\ell _k^t}(u(s_{\ell _k^t})), S(\bar{u}(t))\right) , \end{aligned}$$

which converges to zero as $m \rightarrow \infty$ by (3.43) and Lemma 3.20, where we note that $u(s_{\ell _k^t}) \rightarrow \bar{u}(t)$ as $k \rightarrow \infty$ by (3.44). Since $S(\bar{u}(t))$ is a closed set and the sample path was chosen to be arbitrary, we have that the statement must be true with probability one. $\square$

Now, we show that there is always a strict decrease in $\varphi$ along a trajectory that originates at a noncritical point *z*(0).

#### Lemma 3.23

*Whenever* $z: [0,\infty ) \rightarrow C$ *is a trajectory satisfying the differential inclusion* (3.31) *and* $0 \not \in S(z(0))$*, then there exists a* $T>0$ *such that*

3.45 $$\begin{aligned} \varphi (z(T)) < \sup _{t \in [0,T]} \varphi (z(t)) \le \varphi (z(0)). \end{aligned}$$

#### Proof

We modify the proof from [13, Lemma 5.2]. Let $\delta , \tau$ satisfying $0<\delta <\tau$ be fixed but arbitrary. From Theorem 3.21 we have that *z* is absolutely continuous on $[\delta ,\tau ]$. It is straightforward to show that $\varphi \circ z: [\delta ,\tau ] \rightarrow \mathbb {R}$ is absolutely continuous, since *C* is bounded and $\varphi$ is a composition of a locally Lipschitz map with an absolutely continuous function. Therefore, by Rademacher’s theorem, it is differentiable for almost every $t\in [\delta ,\tau ].$ On the other hand, notice that since $\eta$ is locally Lipschitz near *z*(*t*) and convex, it is Clarke regular, so the chain rule $\partial (\eta \circ z)(t) = \partial \eta (z(t)) \circ \dot{z}(t)$ holds by [11, Theorem 2.3.10]. The chain rule for *j* holds by differentiability. Therefore for almost every *t*, it follows for all $v \in \partial \varphi (z(t))$ that

3.46 $$\begin{aligned} (\varphi \circ z)'(t) = \partial (\varphi \circ z)(t) = (\nabla j(z(t)) + \partial \eta (z(t)))\circ \dot{z}(t) = \langle v, \dot{z}(t) \rangle . \end{aligned}$$

We now observe the following property for the subdifferential of $\delta _C$, namely,

3.47 $$\begin{aligned} \langle v, \dot{z}(t)\rangle =0 \quad \forall v\in N_C(z(t)). \end{aligned}$$

Indeed, since $z(\cdot )$ takes values in *C* and by definition of the subdifferential, for all $r\ge 0$ it follows that

$$\begin{aligned} 0=\delta _C(z(t+r))-\delta _C(z(t)) \ge \langle v, z(t+r)-z(t)\rangle . \end{aligned}$$

Hence,

$$\begin{aligned} 0\ge \lim _{r\rightarrow 0^+} \left\langle v,\frac{z(t+r)-z(t)}{r}\right\rangle = \langle v,\dot{z}(t)\rangle . \end{aligned}$$

The reverse inequality can be obtained by using the left limit of the difference quotient, and we get (3.47). By (3.46) and (3.47), we obtain for a.e. *t* that

3.48 $$\begin{aligned} \langle v, \dot{z}(t)\rangle = \partial (\varphi \circ z)(t) \quad \forall v\in -S(z(t)). \end{aligned}$$

We now show that $\Vert \dot{z}(t) \Vert = d(0,S(z(t)))$. Trivially, $d(0,S(z(t))) \le \Vert \zeta -0\Vert$ for all $\zeta \in S(z(t))$, so it follows that $d(0,S(z(t))) \le \Vert \dot{z}(t)\Vert .$ Notice that for all $v, w \in \partial \varphi (z(t))$, by (3.46), $0 = \langle v-w, \dot{z}(t)\rangle .$ Setting $W := \text {span}(\partial \varphi (z(t)) - \partial \varphi (z(t)))$, we get $\dot{z}(t) \in W^\perp$. Clearly, $-\dot{z}(t) \in (-\dot{z}(t) + W) \cap W^\perp$ so $\Vert \dot{z}(t)\Vert \le d(0, -\dot{z}(t)+W)$. Since $\partial \varphi (z(t)) \subset \dot{z}(t)+W$, it follows $\Vert \dot{z}(t) \Vert \le d(0, \partial \varphi (z(t)))$ and we get $\Vert \dot{z}(t) \Vert = d(0,S(z(t)))$.

Now, notice that by (3.48) and the fact that $\dot{z}(t) \in S(z(t))$, we have for a.e. *t* that

$$\begin{aligned} \partial ({\varphi } \circ z)(t) = -\Vert \dot{z}(t) \Vert ^2 = -d(0,S(z(t)))^2. \end{aligned}$$

Since $\varphi \circ z$ is absolutely continuous on $[\delta ,\tau ]$,

3.49 $$\begin{aligned} \varphi ( z (\tau )) = \varphi ( z (\delta )) - \int _{\delta }^\tau d(0, S(z(s)))^2 \,\mathrm {d}s \end{aligned}$$

and hence $\varphi (z(\delta )) \ge \varphi (z(\tau ))$. Using the continuity of $\varphi \circ z$, and the fact that $0<\delta <\tau$ were arbitrarily chosen, we get $\varphi (z(0)) \ge \varphi (z(t))$ for all $t>0$. To finish the proof, we must find some $T>0$ such that $\varphi (z(T)) < \sup _{t \in [0,T]}\varphi (z(t))$. Suppose that $d(0,S(z(t))) = 0$ for a.e. $t \in [0,T]$ for all $T>0$. Since $\Vert \dot{z}(t) \Vert = d(0,S(z(t)))$ then $z \equiv z(0)$. This is a contradiction, since $\dot{z}(\cdot ) \in S(z(\cdot ))$ and $0 \not \in S(z(0))$. By (3.49), we conclude that there exists a $T>0$ such that (3.45) holds. $\square$

The following proof is standard, but we need to make several arguments differently in the infinite-dimensional setting. We will proceed as in [13]. We define the level sets of $\varphi$ as

$$\begin{aligned} \mathcal {L}_r := \{u\in H: \varphi (u) \le r \}. \end{aligned}$$

#### Proposition 3.24

*For all* $\varepsilon >0$ *there exists a* *N* *such that for all* $n\ge N$*, if* $u_n \in \mathcal {L}_\varepsilon$*, then* $u_{n+1} \in \mathcal {L}_{2 \varepsilon }$ *a.s.*

#### Proof

First, we remark that $\varphi$ is uniformly continuous on *V*, since $\eta (\cdot )$ satisfies (3.17) and, in turn, is Lipschitz continuous on *V*, as well as the fact that *j* is Lipschitz continuous on *V*. Therefore, for any $\varepsilon >0$ there exists a $\delta >0$ such that if $\Vert u_{n+1}- u_n \Vert < \delta$, then $|\varphi (u_{n+1}) - \varphi (u_n)| < \varepsilon .$ Now, we choose *N* such that $\Vert u_{n+1} - u_n \Vert < \delta$ for all $n\ge N$, which is possible by Lemma 3.19. Then it must follow that $|\varphi (u_{n+1}) - \varphi (u_n)| < \varepsilon$ for all $n\ge N$ as well. Now, since $u_n \in \mathcal {L}_\varepsilon$, it follows that $\varphi (u_{n+1}) \le 2\varepsilon$, so therefore $u_{n+1} \in \mathcal {L}_{2\varepsilon }$. $\square$

#### Lemma 3.25

*The following equalities hold.*

3.50 $$\begin{aligned} \liminf _{n \rightarrow \infty } \varphi (u_n) = \liminf _{t\rightarrow \infty } \varphi (u(t)) \quad \text {and} \quad \limsup _{n \rightarrow \infty } \varphi (u_n) = \limsup _{t\rightarrow \infty } \varphi (u(t)). \end{aligned}$$

#### Proof

We argue that $\liminf _{n \rightarrow \infty } \varphi (u_n) \le \liminf _{t\rightarrow \infty } \varphi (u(t))$; the other direction is clear by construction of $u(\cdot )$ from (3.32). Let $\{\tau _n\}$ be a sequence such that $\tau _n \rightarrow \infty$, $\lim _{n \rightarrow \infty } u(\tau _n) = \bar{u}$ for some $\bar{u} \in H$, and $\liminf _{n \rightarrow \infty } \varphi (u(\tau _n)) = \varphi (\bar{u})$. With $k_n := \max \{ n: t_k \le \tau _n\}$, we get

$$\begin{aligned} \Vert u_{k_n} - \bar{u} \Vert \le \Vert u_{k_n} - u(\tau _n) \Vert + \Vert u(\tau _n) - \bar{u} \Vert \le \Vert u_{k_n} - u_{k_{n+1}} \Vert + \Vert u(\tau _n) - \bar{u} \Vert , \end{aligned}$$

which converges to zero as $n\rightarrow \infty$ by (3.24) and convergence of the sequence $\{ u(\tau _n)\}.$ Therefore $u_{k_n} \rightarrow \bar{u}$ and so by continuity of $\varphi$, it follows that

$$\begin{aligned} \liminf _{t \rightarrow \infty } \varphi (u(t)) = \varphi (\bar{u}) = \lim _{n \rightarrow \infty } \varphi (u_{k_n}) \ge \liminf _{n \rightarrow \infty } \varphi (u_n). \end{aligned}$$

Analogous arguments can be made for the claim

$$\begin{aligned} \limsup _{n \rightarrow \infty } \varphi (u_n) = \limsup _{t\rightarrow \infty } \varphi (u(t)). \end{aligned}$$

$$\square$$

#### Lemma 3.26

*Only finitely many iterates* $\{ u_n\}$ *are* *contained in* $H \backslash \mathcal {L}_{2\varepsilon }.$

#### Proof

We choose $\varepsilon >0$ such that $\varepsilon \notin \varphi (S^{-1}(0)),$ which is possible for arbitrarily small $\varepsilon$ by Assumption 3.11(v), where we note that $\varphi (S^{-1}(0)) = f(S^{-1}(0))$. We construct the process given by the recursion

$$\begin{aligned} i_1&:= \min \{n : u_n \in \mathcal {L}_\varepsilon \text { and } u_{n+1}\in \mathcal {L}_{2\varepsilon } \backslash \mathcal {L}_{\varepsilon }\},\\ e_1&:= \min \{ n: n> i_1 \text { and } u_n \in H\backslash \mathcal {L}_{2\varepsilon }\}, \\ i_2&:= \min \{n: n > e_1 \text { and } u_n \in \mathcal {L}_\varepsilon \}, \end{aligned}$$

and so on. We argue by contradiction and recall that $s_n = \sum _{j=1}^{n-1} t_j$. Suppose infinitely many $\{ u_n\}$ are in $H \backslash \mathcal {L}_{2\varepsilon }$, then it must follow that $i_j \rightarrow \infty$ as $j \rightarrow \infty$. By Theorem 3.22, $\{u(\cdot +s_{i_j})\}$ is relatively compact in *C*([0, *T*], *H*) for all $T>0$ and there exists a subsequence (with the same labeling) and limit point $z(\cdot )$ such that $z(\cdot )$ is a trajectory of (3.31). Now, since by construction $\varphi (u_{i_j}) \le \varepsilon$ and $\varphi (u_{i_j+1}) > \varepsilon$, it follows that

3.51 $$\begin{aligned} \begin{aligned} \varepsilon \ge \varphi (u_{i_j})& = \varphi (u_{i_j+1}) + \varphi (u_{i_j}) - \varphi (u_{i_j+1})\\&\ge \varepsilon + \varphi (u_{i_j})- \varphi (u_{i_j+1}). \end{aligned} \end{aligned}$$

Recall that $\lim _{j \rightarrow \infty } u_{i_j} = u(\cdot +s_{i_j}) = z(0)$. Taking the limit $j \rightarrow \infty$ on both sides of (3.51), by continuity of $\varphi$, we get

$$\begin{aligned} \lim _{j \rightarrow \infty } \varphi (u_{i_j}) = \varphi (z(0)) = \varepsilon , \end{aligned}$$

meaning *z*(0) is not a critical point of $\varphi$. Thus we can invoke Lemma 3.23 to get the existence of a $T>0$ such that

3.52 $$\begin{aligned} \varphi (z(T)) < \sup _{t \in [0,T]} \varphi (z(t)) \le \varphi (z(0)) = \varepsilon . \end{aligned}$$

By uniform convergence of $u(\cdot +s_{i_j})$ to $z(\cdot )$, it follows for *j* sufficiently large that

$$\begin{aligned} \sup _{t \in [0,T]} |\varphi (u(t+s_{i_j})) - \varphi (z(t))| < \varepsilon , \end{aligned}$$

so

$$\begin{aligned} \sup _{t \in [0,T]} \varphi (u(t+s_{i_j})) \le \sup _{t \in [0,T]} |\varphi (u(t+s_{i_j})) - \varphi (z(t))| + \sup _{t \in [0,T]} \varphi (z(t)) \le 2\varepsilon . \end{aligned}$$

Therefore it must follow that

3.53 $$\begin{aligned} s_{e_{i_j}} > s_{i_j} + T \end{aligned}$$

for *j* sufficiently large. We now find a contradiction to the statement (3.53). This is done by observing the sequence $\ell _j := \max \{ \ell : s_{i_j} \le s_\ell \le s_{i_j} + T\}.$ From (3.52), we have that there exists a $\delta > 0$ such that $\varphi (z(T)) \le \varepsilon - 2 \delta .$ Observe that

$$\begin{aligned} \Vert u_{\ell _j} - u(T+s_{i_j}) \Vert = \Vert u(s_{\ell _j}) - u(T+s_{i_j})\Vert \le \Vert u_{\ell _j} - u_{\ell _j + 1}\Vert \rightarrow 0 \quad \text { as } j \rightarrow \infty . \end{aligned}$$

Therefore $u_{\ell _j} \rightarrow u(T+s_{i_j})$ and hence $u_{\ell _j} \rightarrow z(T)$ as $j \rightarrow \infty$. By continuity, we get $\lim _{j \rightarrow \infty } \varphi (u_{\ell _j}) = \varphi (z(T)).$ Thus $\varphi (u_{\ell _j}) < \varepsilon - \delta$ for *j* sufficiently large, a contradiction to (3.53). $\square$

#### Proposition 3.27

*The limit* $\lim _{t \rightarrow \infty } \varphi (u(t))$ *exists.*

#### Proof

W.l.o.g. assume $\liminf _{t\rightarrow \infty }\varphi (u(t))=0$; this is possible by the fact that *j* and $\eta$ are bounded below. Choosing $\varepsilon >0$ such that $\varepsilon \notin \varphi (S^{-1}(0)),$ we have by Lemma 3.26 that for *N* sufficiently large, $u_n \in \mathcal {L}_{2\varepsilon }$ for all $n \ge N$. Since $\varepsilon$ can be chosen to be arbitrarily small, we conclude that $\lim _{t\rightarrow \infty } \varphi (u(t)) = 0.$ $\square$

#### Proof of Theorem 3.13

The fact that $\{\varphi (u_n)\}$ converges follows from Proposition 3.27 and Lemma 3.25. Since $\{u_n \} \subset C$, it trivially follows that $\{ f(u_n)\}$ converges a.s. Let $\bar{u}$ be a limit point of $\{ u_n\}$ and suppose that $0 \notin S(\bar{u})$. Let $\{ u_{n_k}\}$ be a subsequence converging to $\bar{u}$ and let $z(\cdot )$ be the limit of $\{u(\cdot +s_{n_k})\}$. Then, by Lemma 3.23, there exists a $T>0$ such that

3.54 $$\begin{aligned} \varphi (z(T)) < \sup _{t \in [0,T]} \varphi (z(t)) \le \varphi (\bar{u}). \end{aligned}$$

However, it follows from Proposition 3.27 that

$$\begin{aligned} \varphi (z(T)) = \lim _{k \rightarrow \infty } \varphi (u(T+s_{n_k})) = \lim _{t\rightarrow \infty } \varphi (u(t)) = \varphi (\bar{u}), \end{aligned}$$

which is a contradiction to (3.54). $\square$

## Application to PDE-constrained optimization under uncertainty

In this section, we apply the algorithm presented in Sect. 3.2 to a nonconvex problem from PDE-constrained optimization under uncertainty. In Sect. 4.1, we set up the problem and verify conditions for convergence of the stochastic proximal gradient method. We show numerical experiments in Sect. 4.2.

### Model problem

We first introduce notation and concepts specific to our application; see [18, 52]. Let $D \subset \mathbb {R}^d$, $d \le 3$ be an open and bounded Lipschitz domain. The inner product between vectors $x, y \in \mathbb {R}^d$ is denoted by $x \cdot y = \sum _{i=1}^d x_i y_i$. For a function $v:\mathbb {R}^d \rightarrow \mathbb {R}$, let $\nabla v(x) = ({\partial v(x)}/{\partial x_1}, \dots , {\partial v(x)}/{\partial x_d})^\top$ denote the gradient and for $w: \mathbb {R}^d \rightarrow \mathbb {R}^d$, let $\nabla \cdot w(x) = {\partial w_1(x)}/{\partial x_1} + \cdots + {\partial w_d(x)}/{\partial x_d}$ denote the divergence. We define the Sobolev space $H^1(D)$ = {$u\in L^2(D)$ having weak derivatives ${\partial u}/{\partial x_i} \in L^2(D)$, $i =1, \dots , d$} and the closure of $C_c^\infty (D)$ in $H^1(D)$ by $H_0^1(D)$.

We will focus on a semilinear diffusion-reaction equation with uncertainties, which describes transport phenomena at equilibrium and is motivated by [41]. We assume that there exist random fields $a: D \times \varOmega \rightarrow \mathbb {R}$ and $r: D \times \varOmega \rightarrow \mathbb {R}$, which are the diffusion and reaction coefficients, respectively. To facilitate simulation, we will make a standard finite-dimensional noise assumption, meaning the random field has the form

$$\begin{aligned} a(x,\omega ) = a(x,\xi (\omega )), \quad r(x,\omega ) = r(x,\xi (\omega )) \quad \text { in } D \times \varOmega , \end{aligned}$$

where $\xi (\omega ) = (\xi _1(\omega ), \dots , \xi _m(\omega ))$ is a vector of real-valued uncorrelated random variables $\xi _{i}:\varOmega \rightarrow \varXi _i \subset \mathbb {R}$. The support of the random vector will be denoted by $\varXi := \prod _{i=1}^m \varXi _i$. We consider the following PDE constraint, to be satisfied for almost every $\xi \in \varXi$:

4.1 $$\begin{aligned} \begin{aligned} - \nabla \cdot (a(x,\xi ) \nabla y(x,\xi )) + r(x,\xi ) (y(x,\xi ))^3& = u(x), \qquad (x,\xi ) \in D \times \varXi , \\ y(x,\xi )& = 0, \quad \qquad (x,\xi ) \in \partial D \times \varXi .\\ \end{aligned} \end{aligned}$$

Optimal control problems with semilinear PDEs involving random coefficients have been studied in, for instance, [26, 27]. We include a nonsmooth term as in [14] with the goal of obtaining sparse solutions. In the following, we assume that $\lambda _1 \ge 0$, $\lambda _2 \ge 0$, and $y_D \in L^2(D)$. The model problem we solve is given by

P' $$\begin{aligned} \begin{aligned}&\min _{u \in C} \quad \left\{ \varphi (u):= \frac{1}{2} \mathbb {E}[ \Vert y(\xi ) - y_D\Vert _{L^2(D)}^2 ] + \frac{\lambda _2}{2} \Vert u \Vert _{L^2(D)}^2 +\lambda _1 \Vert u \Vert _{L^1(D)}\right\} \\&\quad \text {s.t.} \quad - \nabla \cdot (a(x,\xi ) \nabla y) + r(x,\xi ) y^3 = u(x), \qquad (x,\xi ) \in D \times \varXi , \\&\qquad\qquad\qquad\qquad\qquad\qquad\quad y = 0, \quad \qquad (x,\xi ) \in \partial D \times \varXi ,\\&\quad \quad \quad \quad \quad C:= \{ u \in L^2(D): \,u_a(x) \le u(x) \le u_b(x)\,\,\text { a.e. } x\in D\}. \end{aligned} \end{aligned}$$

The following assumptions will apply in this section. In particular, we do not require uniform bounds on the coefficient $a(\cdot ,\xi )$, which allow for modeling with log-normal random fields.

#### Assumption 4.1

We assume $y_D \in L^2(D)$, $u_a, u_b \in L^2(D)$, and $u_a \le u_b$. There exist $a_{\min }(\cdot ), a_{\max }(\cdot )$ such that $0< a_{\min }(\xi )< a(\cdot ,\xi )< a_{\max }(\xi )< \infty$ in *D* a.s. and $a_{\min }^{-1}, a_{\max } \in L^p(\varXi )$ for all $p \in [1,\infty )$. Furthermore, there exists $r_{\max }(\cdot )$ such that $0 \le r(\cdot ,\xi ) \le r_{\max }(\xi )<\infty$ a.s. and $r_{\max } \in L^p(\varXi )$ for all $p \in [1,\infty )$.

Existence of a solution to Problem (P’) follows by applying [27, Proposition 3.1]. The following result holds by [27, Proposition 2.1] combined with standard a priori estimates for a fixed realization $\xi$ to obtain (4.2) and (4.3).

#### Lemma 4.2

*For almost every* $\xi \in \varXi$, (4.1) *has a unique solution* $y(\xi )=y(\cdot ,\xi ) \in H_0^1(D)$ *and there exists a positive random variable* $C_1 \in L^p(\varXi )$ *for all* $p \in [1,\infty )$ *independent of* *u* *such that for almost every* $\xi \in \varXi$,

4.2 $$\begin{aligned} \Vert y(\xi ) \Vert _{L^2(D)} \le C_1(\xi ) \Vert u \Vert _{L^2(D)}. \end{aligned}$$

*Additionally, for* $y_1(\xi )$ *and* $y_2(\xi )$ *solving* (4.1) *with* $u=u_1$ *and* $u=u_2$*, respectively, we have for almost every* $\xi \in \varXi$ *that*

4.3 $$\begin{aligned} \Vert y_1(\xi ) - y_2(\xi ) \Vert _{L^2(D)} \le C_1(\xi ) \Vert u_1 - u_2 \Vert _{L^2(D)}. \end{aligned}$$

By Lemma 4.2, the control-to-state operator $T(\xi ):L^2(D) \rightarrow H_0^1(D), u \mapsto T(\xi )u$ is well-defined for almost every $\xi$ and all $u \in L^2(D)$. Additionally, for almost every $\xi \in \varXi$, this mapping is in fact continuously Fréchet differentiable; this can be argued by verifying [23, Assumption 1.47] as in [23, pp. 76-78]. With that, we define the reduced functional $J:L^2(D) \times \varXi \rightarrow \mathbb {R}$ by $J(u,\xi ):= \frac{1}{2} \Vert T(\xi )u - y_D\Vert _{L^2(D)}^2 + \frac{\lambda _2}{2} \Vert u \Vert _{L^2(D)}^2$ and we can define the stochastic gradient.

#### Proposition 4.3

$J:L^2(D)\times \varXi \rightarrow \mathbb {R}$ *is continuously* *Fréchet* *differentiable and the stochastic gradient is given by*

4.4 $$\begin{aligned} G(u,\xi ) := \lambda _2 u - p(\cdot ,\xi ), \end{aligned}$$

*where, given a solution* $y = y(\cdot ,\xi )$ *to* (4.1)*, the function* $p = p(\cdot ,\xi ) \in H_0^1(D)$ *is the solution to the adjoint equation*

4.5 $$\begin{aligned} \begin{aligned} -\nabla \cdot (a(x,\xi ) \nabla p) + 3 r(x, \xi )y^2 p& = y_D-y, \quad (x,\xi ) \in D \times \varXi \\ p& =0, \qquad \quad (x,\xi ) \in \partial D \times \varXi . \end{aligned} \end{aligned}$$

*Furthermore, for almost every* $\xi \in \varXi$, *with the same* $C_1 \in L^p(\varXi )$ *for all* $p \in [1,\infty )$ *as in Lemma* 4.2,

4.6 $$\begin{aligned} \Vert p(\cdot ,\xi ) \Vert _{L^2(D)} \le C_1(\xi ) \Vert y_D - y(\xi )\Vert _{L^2(D)}. \end{aligned}$$

*Additionally, for* $p_1(\xi )$ *and* $p_2(\xi )$ *solving* (4.5) *with* $y=y_1(\xi )$ *and* $y=y_2(\xi )$, *respectively (where *$y_i(\xi )$ *solves* (4.1) *with* $u=u_i$*),*

4.7 $$\begin{aligned} \Vert p_1(\xi ) - p_2(\xi ) \Vert _{L^2(D)} \le C_1(\xi ) \Vert y_1(\xi ) - y_2(\xi ) \Vert _{L^2(D)}. \end{aligned}$$

The proofs of the above and following proposition are in Sect. B. We define $j:L^2(D) \rightarrow \mathbb {R}$ by $j(u):= \mathbb {E}[J(u,\xi )]$ for all $u\in L^2(D)$ and show that it is continuously Fréchet differentiable in the following proposition.

#### Proposition 4.4

*The function* $j:L^2(D) \rightarrow \mathbb {R}$ *is continuously Fréchet differentiable and* $\mathbb {E}[G(u,\xi )] = \nabla j(u)$ *for all* $u\in L^2(D)$.

Now, we present the main result of this section, which is the verification of assumptions for the convergence of Algorithm 2.

#### Theorem 4.5

*Problem (P’) satisfies Assumption* 3.1(ii) *as well as Assumptions* 3.11(i)–3.11(iv).

#### Proof

For Assumption 3.1(ii), we note that by Proposition 4.4, *j* is continuously Fréchet differentiable and $\mathbb {E}[G(u,\xi )] = \nabla j(u)$ for all $u \in L^2(D)$. Now, for arbitrary $u_1,u_2 \in L^2(D)$, we have by Jensen’s inequality, (4.4), and Hölder’s inequality applied to (4.7) and (4.3) that

$$\begin{aligned}&\Vert \nabla j(u_1) - \nabla j(u_2)\Vert _{L^2(D)} \le \mathbb {E}[\Vert G(u_1,\xi )-G(u_2,\xi )\Vert _{L^2(D)}]\\&\qquad \quad\le \lambda _2 \Vert u_1 - u_2 \Vert _{L^2(D)} + \mathbb {E}[\Vert p_1(\xi ) - p_2(\xi )\Vert _{L^2(D)} ]\\&\quad \qquad \le \lambda _2 \Vert u_1 - u_2 \Vert _{L^2(D)} + \left( \mathbb {E}[(C_1(\xi ))^2]\right) ^{1/2}\left( \mathbb {E}[\Vert y_1(\xi ) - y_2(\xi )\Vert _{L^2(D)}^2 ]\right) ^{1/2}\\& \qquad \quad \le \lambda _2 \Vert u_1 - u_2 \Vert _{L^2(D)} + \Vert C_1\Vert _{L^2(\varXi )}^2 \Vert u_1 - u_2\Vert _{L^2(D)}. \end{aligned}$$

Since $\Vert C_1\Vert _{L^2(\varXi )}^2 < \infty$ it follows that $j \in C^{1,1}_L(L^2(D))$.

Assumption 3.11(i) is obviously satisfied. For Assumption 3.11(ii), we have that the function $\eta (u)=\lambda _1 \Vert u\Vert _{L^1(D)} \in \varGamma _0(L^2(D))$ and is clearly bounded below; additionally, $\eta$ is globally Lipschitz and therefore satisfies (3.17). For Assumption 3.11(iii), we have by (4.4), (4.6), and (4.2) the bound

4.8 $$\begin{aligned} \Vert G(u,\xi ) \Vert _{L^2(D)} \le \lambda _2 \Vert u \Vert _{L^2(D)} + C_1(\xi ) \Vert y_D \Vert _{L^2(D)} + (C_1(\xi ))^2 \Vert u\Vert _{L^2(D)} \end{aligned}$$

and furthermore $\mathbb {E}[\Vert G(u,\xi ) \Vert _{L^2(D)}^2] =:M(u)<\infty$ by integrability of $\xi \mapsto C_1(\xi )$. Assumption 3.11(iv) follows for any $u \in C$ (and hence any convergent sequence $\{u_n\}$ in *C*) by (4.8). $\square$

The last assumption from Assumption 3.11 is technical and difficult to verify for general functions in infinite dimensions. Indeed, [31] gave an example of a $C^\infty$-function whose critical values make up a set of measure greater than zero. In finite dimensions the story is easier: the Morse–Sard theorem guarantees that Assumption 3.11(v) holds if $f:\mathbb {R}^n \rightarrow \mathbb {R}$ and $f \in C^k$ for $k \ge n$. In infinite dimensions, certain well-behaved functions, in particular Fredholm operators, see [50], satisfy this assumption.

### Numerical experiments

In this section, we demonstrate Algorithm 2 on Problem (P’). Simulations were run using FEniCS by [2] on a laptop with Intel Core i7 Processor (8 x 2.6 GHz) with 16 GB RAM. Let the domain be given by $D=(0,1)\times (0,1)$ and the constraint set be given by $C= \{ u \in L^2(D) \,|\, -0.5 \le u(x) \le 0.5 \,\, \forall x \in D\}.$ We modify [14, Example 6.1], with $y_D(x)=\sin (2 \pi x_1)\sin (2\pi x_2) \exp (2 x_1)/6$, $\lambda _1 = 0.008$, and $\lambda _2 = 0.001.$ We generate random fields using a Karhunen-Loève expansion, with means $a_0 = 0.5$ and $r_0 = 0.5$, number of summands $m = 20$, and $\xi ^{a,i},\xi ^{r,i} \sim U(-\sqrt{0.5},\sqrt{0.5})$, where *U*(*a*, *b*) denotes the uniform distribution between real numbers *a* and *b*, $a<b$. The eigenfunctions and eigenvalues are given by

$$\begin{aligned} \tilde{\phi }_{j,k}(x):= 2\cos (j \pi x_2)\cos (k \pi x_1), \quad \tilde{\lambda }_{k,j}:=\frac{1}{4} \exp (-\pi (j^2+k^2)l^2), \quad j,k \ge 1, \end{aligned}$$

where we reorder terms so that the eigenvalues appear in descending order (i.e., $\phi _1 = \tilde{\phi }_{1,1}$ and $\lambda _1 = \tilde{\lambda }_{1,1}$) and we choose correlation length $l=0.5$. Thus

4.9 $$\begin{aligned} a(x,\xi ) = a_0 + \sum _{i=1}^m \sqrt{\lambda _i} \phi _i \xi ^{a,i}, \quad r(x,\xi ) = r_0 + \sum _{i=1}^m \sqrt{\lambda _i} \phi _i \xi ^{r,i}. \end{aligned}$$

For Algorithm 2, we generate samples with $\xi _n = (\xi _n^{a,1}, \dots , \xi _n^{a,m}, \xi _n^{r,a}, \dots ,\xi _n^{r,m})$ at each iteration *n*. The step size is chosen to be $t_n = \theta /n$ with $\theta = 100$, where the scaling was chosen such that $\theta \approx 1/\Vert G(u_1, \xi _1)\Vert$. The initial point was $u_1(x) = \sin (4\pi x_1) \sin (4 \pi x_2).$

A uniform mesh $\mathcal {T}$ with 9800 shape regular triangles *T* was used. We denote the mesh fineness with $\hat{h} = \max _{T \in \mathcal {T}}{\text {diam}}(T)$. The state and adjoint were discretized using piecewise linear finite elements, (where $\mathcal {P}_i$ denotes the space of polynomials of degree up to *i*), given by the set

$$\begin{aligned} V_{\hat{h}}&:= \lbrace v \in H_0^1(D): v|_{T} \in \mathcal {P}_1(T) \text { for all } T\in \mathcal T \rbrace . \end{aligned}$$

For the controls, we choose a discretization of $L^2(D)$ by piecewise constants, given by the set

$$\begin{aligned} U_{\hat{h}}&:= \lbrace u \in L^2(D): v|_{T} \in \mathcal {P}_0(T) \text { for all } T\in \mathcal {T} \rbrace ,\quad C_{\hat{h}} := U_{\hat{h}}\cap C. \end{aligned}$$

We use the $L^2$-projection $P_{\hat{h}}:L^2(D) \rightarrow U_{\hat{h}}$ defined for each $v \in L^2(D)$ by

$$\begin{aligned} P_{\hat{h}}(v)\bigl |_T := \frac{1}{|T|}\int _T v\,\mathrm {d}x. \end{aligned}$$

This is done to project the stochastic gradient onto the $L^2(D)$ space as in [20]. Hence, the last line of Algorithm 2 is given by the expression $u_{n+1}:=\text {prox}_{t_n h}\left( u_n - t_nP_{\hat{h}} G(u_n,\xi _{n})\right) .$ For the computation of the proximity operator $\text {prox}_{t(\eta +\delta _C)}(z) = {{\,\mathrm{arg\,min}\,}}_{-0.5 \le v \le 0.5} \{ \lambda _1 \Vert v \Vert _{L^1(D)} + \frac{1}{2t} \Vert v - z \Vert _{L^2(D)}^2\}$, we use the formula from [5, Example 6.22], defined piecewise on each element of the mesh. For each $T \in \mathcal {T}$, it is given by

$$\begin{aligned} \text {prox}_{t(\eta +\delta _C)}(z|_T) = \min \{ \max \{ |z|_T|-t \lambda _1,0\},0.5\}\mathrm{sgn} (z|_T). \end{aligned}$$

For convergence plots, we use a heuristic to approximate the objective function and the measure of stationarity by increasing sampling as the control reaches stationarity. To be more precise, we use a sequence of sample sizes $\{m_n\}$ with $m_{ n} = 10\lfloor \tfrac{n}{50}\rfloor +1$ newly generated i.i.d. samples $(\xi _{n,1}, \dots , \xi _{n,m_n})$ and compute

$$\begin{aligned} \hat{f}_n&:= \frac{1}{m_n}\sum _{j=1}^{m_n} J(u_n, \xi _{n,j}) + \eta (u_n), \\ {r}_n&:= \left\Vert u_n - \text {prox}_{\eta +\delta _C}\left( u_n - \frac{1}{m_n}\sum _{j=1}^{m_n}P_{\hat{h}} G(u_n,\xi _{n,j})\right) \right\Vert _{L^2(D)}. \end{aligned}$$

**Fig. 1:**
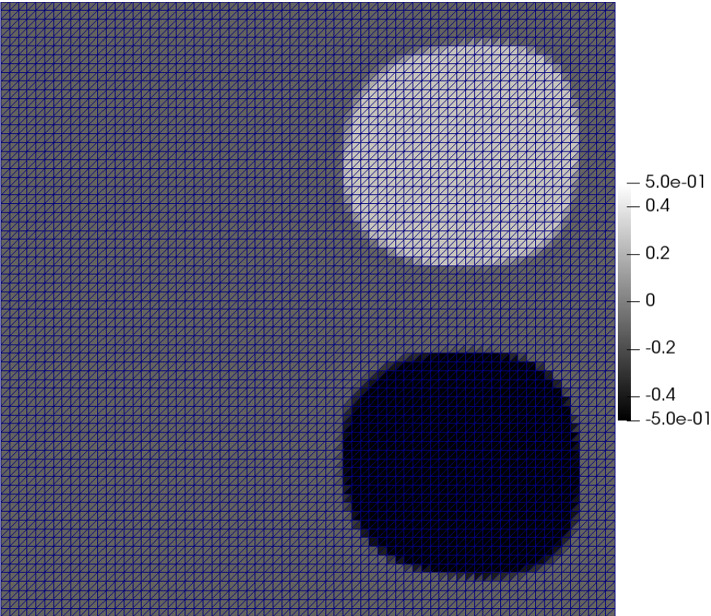
The control *u* after 251 iterations

**Fig. 2:**
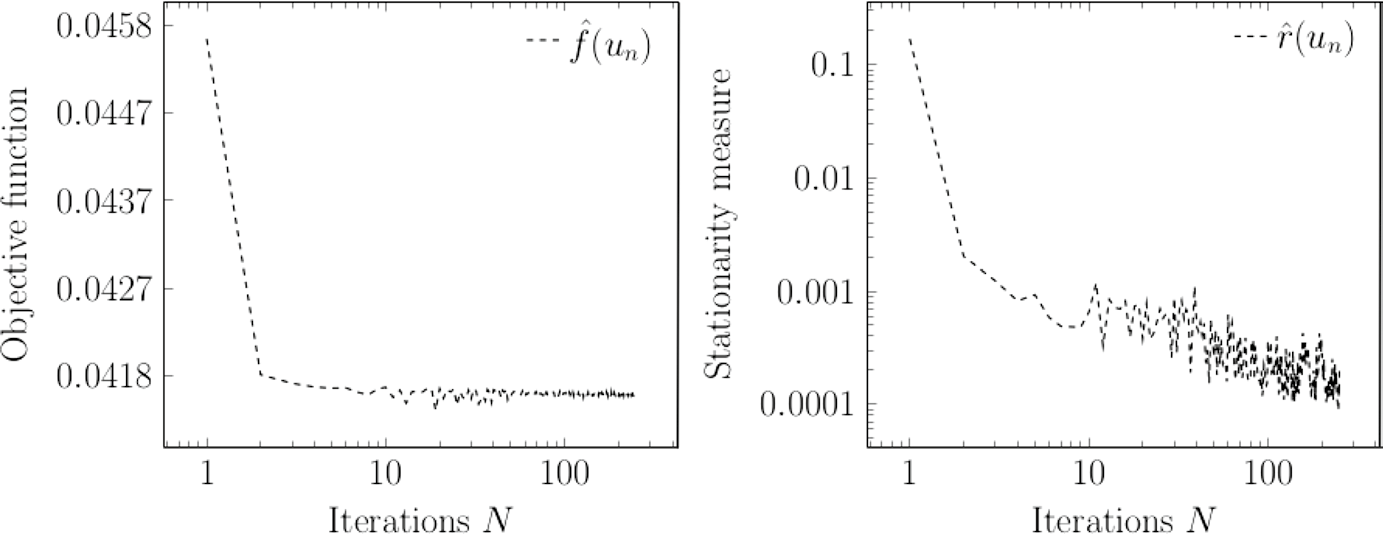
Behavior of the objective function (left) and the stationarity measure (right)

The algorithm is terminated for $n\ge 50$ if $\hat{r}_n := \sum _{k=n-50}^n {r}_n \le \text {tol}$ with $\text {tol}=2e^{-4}$. The parameters for our heuristic termination rule were tuned, for illustration purposes only, so that the algorithm stopped after several hundred iterations. A plot of the control after termination is shown in Fig. 1. The effect of the sparse term $\eta$ as well as the constraint set *C* can be seen clearly. Decay of the objective function value and the stationarity measure are shown in Fig. 2. We see convergence of the objective function values and the stationarity measure tends to zero as expected.

Additionally, we conduct an experiment to demonstrate mesh independence of the algorithm by running the algorithm once each for different meshes and comparing the number of iterations needed until the tolerance $\text {tol}$ is reached. In Table 1, we see that these iteration numbers are of the same order. The estimate for the objective function $\hat{f}_N$ is also included at the final iteration *N*, demonstrating how solutions become more exact on finer meshes.

**Table 1:** Experiment showing mesh independence

$\hat{h}$	# triangles	Objective function $\hat{f}_N$	# iterations *N* until $\hat{r}_N \le$ tol
7.1e$^{-2}$	800	4.160e$^{-2}$	191
4.7e$^{-2}$	1800	4.157e$^{-2}$	295
3.5e$^{-2}$	3200	4.157e$^{-2}$	233
2.8e$^{-2}$	5000	4.156e$^{-2}$	257
2.4e$^{-2}$	7200	4.156e$^{-2}$	271
2.0e$^{-2}$	9800	4.155e$^{-2}$	251

## Conclusion

In this paper, we presented asymptotic convergence analysis for two variants of the stochastic proximal gradient algorithm in Hilbert spaces. The main results address the asymptotic convergence to stationary points of general functions defined over a Hilbert space. Moreover, we presented an application to the theory in the form of a problem from PDE-constrained optimization under uncertainty. Assumptions for convergence were verified for a tracking-type problem with a $L^1$-penalty term subject to a semilinear elliptic PDE with random coefficients and box constraints. Numerical experiments demonstrated the effectiveness of the method.

The ODE method from Sect. 3.2 allowed us to prove a more general result with weaker assumptions on the objective function. However, we needed to introduce an assumption on the set of critical values in the form of Assumption 3.11(v). While we did not verify this assumption for our model problem, it would be interesting to know whether this assumption is verifiable for this class of problems. We had to be slightly more restrictive on the nonsmooth term in Sect. 3.2 than we were in Sect. 3.1. The advantages in terms of computational cost of Algorithm 2 over Algorithm 1 are clear: the use of decreasing step sizes in Algorithm 2 means that increased sampling is not needed. Additionally, there is no need to determine the Lipschitz constant for the gradient, which in the application depends on (among other things) the Poincaré constant and the lower bound on the random fields, and thus lead to a prohibitively small constant step size. This phenomenon has been demonstrated in [20].

How to scale the decreasing step size $t_n$ remains an open question. In practice, the scaling of the step size can be tuned offline. An improper choice of the scaling *c* in the step size $t_n = c/n^\alpha$ for $0.5 < \alpha \le 1$ can lead to arbitrarily slow convergence; this was demonstrated in [39]. While this was not the focus of our work, efficiency estimates for nonconvex problems might also be possible following the work by [7, 21, 35]. In lieu of efficiency estimates, it would be desirable to have better termination conditions that do not rely on increased sampling as our heuristic did in the numerical experiments. Finally, it would be natural to investigate mesh refinement strategies as in [20]. For more involved choices of nonsmooth terms, the $\text {prox}$ computation is also subject to numerical error and should be treated.

## A Auxiliary results

To prove Lemma 3.17, we first need the following result.

### Proposition A.1

*For* $1 \le p \le \infty$*, every* *H**-valued martingale that is bounded in the Bochner space* $L^p(\varOmega ,H)$ *converges a.s.*

### Proof

Since *H* is a Hilbert space, it is reflexive and therefore has the Radon–Nikodym property by [43, Corollary 2.11]. The rest of the proof can be found in [43, Theorem 2.5]. $\square$

### Proof of Lemma 3.17

It is straightforward to show that $\mathbb {E}[\Vert v_n\Vert ^2] = \mathbb {E}[\Vert v_1 \Vert ^2] + \sum _{k=1}^{n-1} \mathbb {E}[\Vert v_{k+1} - v_{k}\Vert ^2],$ and therefore boundedness of $v_n$ for all *n* follows from (3.22) and vice versa. Supposing now that (3.22) holds, the fact that $\{v_n\}$ converges to a limit $v_\infty$ follows by Proposition A.1. $\square$

## B Auxiliary proofs for application

### Proof of Proposition 4.3

Continuous differentiability of $J(\cdot ,\xi ):L^2(D)\rightarrow \mathbb {R}$ follows from continuous differentiability of $u \mapsto T(\xi )u$ and the fact that $(u,y) \mapsto \tilde{J}(u,y) = \tfrac{1}{2} \Vert y-y_D\Vert _{L^2(D)}^2 + \tfrac{\lambda _2}{2}\Vert u\Vert _{L^2(D)}^2$ is continuously Fréchet differentiable. One obtains (4.4) and (4.5) by fixing a realization $\xi \in \varXi$ and computing the derivative of $u \mapsto J(u,\xi )$ as in, e.g., [23, pp. 58-59]. Bounds (4.6) and (4.7) follow from standard a priori estimates. $\square$

### Proof of Proposition 4.4

We verify the conditions of Lemma C.3 from Sect. C. Fréchet differentiability of $J:L^2(D) \times \varXi \rightarrow \mathbb {R}$ for almost every $\xi$ follows from Proposition 4.3. The function *j* is well-defined and finite-valued for all $u \in L^2(D)$, since

$$\begin{aligned} j(u)& = \frac{1}{2}\mathbb {E}[\Vert y - y_D \Vert ^2_{L^2(D)}] + \frac{\lambda _2}{2} \Vert u \Vert _{L^2(D)}^2 \le \mathbb {E}[\Vert T(\xi )u \Vert _{L^2(D)}^2] + \Vert y_D\Vert _{L^2(D)}^2 + \frac{\lambda _2}{2} \Vert u \Vert _{L^2(D)}^2 \end{aligned}$$

is finite by $T(\xi )u = y(\xi )$ and (4.2) along with the assumption that $y_D \in L^2(D)$. Now, for every $v \in C$, there exists a $y_v(\xi )$ satisfying (4.1) with $u=v$ and a $p_v(\xi )$ satisfying (4.5) with $y=y_v(\xi )$. Thus by (4.6) followed by (4.2),

$$\begin{aligned} \Vert G(v,\xi ) \Vert _{L^2(D)}& = \Vert \lambda _2v - p_v(\xi ) \Vert _{L^2(D)} \le \lambda _2 \Vert v \Vert _{L^2(D)} + C_1(\xi )\Vert y_D - y_v(\xi )\Vert _{L^2(D)}\\&\le \lambda _2 \Vert v \Vert _{L^2(D)} + C_1(\xi ) \Vert y_D \Vert _{L^2(D)} + (C_1(\xi ))^2 \Vert v\Vert _{L^2(D)}=:C(\xi ). \end{aligned}$$

Notice that $C \in L^p(\varXi )$ for all $p \in [1,\infty )$ by nature of the mapping $\xi \mapsto C_1(\xi )$. Therefore, the conditions of Lemma C.3 are satisfied and we have proven Fréchet differentiability of *j*. $\square$

## C Differentiability of expectation functionals

Let $(X, \Vert \cdot \Vert _X)$ be a Banach space and let $J: X \times \varOmega \rightarrow \mathbb {R}$ be a random variable functional. We summarize under what conditions we can exchange the integral and the derivative for the functional $j: X \rightarrow \mathbb {R}$, where $j(u) = \int _\varOmega J(u,\omega ) \,\mathrm {d}\mathbb {P}(\omega )$.

The following definition gives the minimal requirement for exchanging the derivative and expectation, namely, requiring $J:X \times \varOmega \rightarrow \mathbb {R}$ to be $L^1$-Fréchet differentiable.

### Definition C.1

A *p*-times integrable random functional $J:X \times \varOmega \rightarrow \mathbb {R}$ is called $L^p$-Fréchet differentiable at *u* if for an open set $U \subset X$ there exists a bounded and linear random operator $A:U \times \varOmega \rightarrow \mathbb {R}$ such that $\lim _{h \rightarrow 0} \Vert J_{\omega }(u + h) - J_{\omega }(u) + A(u,\omega )h \Vert _{L^p(\varOmega )} / \Vert h \Vert _X = 0$.

By Hölder’s inequality, if $u \mapsto J(u,\cdot )$ is $L^p$-differentiable and $1 \le r < p$, then it is also $L^r$-differentiable with the same derivative. This implies that $j:X \rightarrow \mathbb {R}$ is Fréchet differentiable at *u*.

The condition in Definition C.1 might be difficult to verify directly. For this reason, we consider other assumptions on an open neighborhood *U* of *X* containing *u*. We denote the functional $J(\cdot ,\omega ):X \rightarrow \mathbb {R}$ for a fixed realization $\omega \in \varOmega$ by $J_\omega :X \rightarrow \mathbb {R}$.

### Assumption C.2

(vi) The expectation *j*(*v*) is well-defined and finite-valued for all $v \in U.$
(vii) For almost every $\omega \in \varOmega$, the functional $J_\omega :X \rightarrow \mathbb {R}$ is Fréchet differentiable at *u*. Moreover, there exists a positive random variable $C(\cdot ) \in L^1(\varOmega )$ such that for all $v \in U$ and almost every $\omega \in \varOmega$,
  C.1 $$\begin{aligned} \Vert J'_\omega (v) \Vert _{X^*} \le C(\omega ). \end{aligned}$$

### Lemma C.3

*Suppose Assumption* C.2*holds. Then* *j* *is Fréchet differentiable at* *u* *and*

C.2 $$\begin{aligned} j'(u) = \mathbb {E}[J'_\omega (u)]. \end{aligned}$$

### Proof

By the mean value theorem, for *h* close enough to *u*, there exists a *z* within the neighborhood containing $u+h$ and *u* that satisfies $| J_{\omega }(u + h) - J_{\omega }(u) | \le \Vert J'_\omega (z)\Vert _{X^*} \Vert h\Vert _{X}.$ Now, we have for almost every $\omega \in \varOmega$ that

$$\begin{aligned} \begin{aligned} \frac{|J_{\omega }(u+h) - J_{\omega }(u) - J'_{\omega }(u)h|}{\Vert h \Vert _X}&\le \frac{|J_{\omega }(u+h) - J_{\omega }(u) |}{\Vert h \Vert _X} + \frac{|J'_{\omega }(u)h|}{\Vert h \Vert _X}\\&\le \Vert J'_{\omega }(z) \Vert _{X^*} + \Vert J'_{\omega }(u) \Vert _{X^*} \le 2 C(\omega ). \end{aligned} \end{aligned}$$

By Assumption C.2(vii), $C(\cdot )$ is integrable, so by Lebesgue’s dominated convergence theorem, it follows that

C.3 $$\begin{aligned} \begin{aligned}&\lim _{h \rightarrow 0} \frac{\int _{\varOmega } |J_{\omega }(u+h) - J_{\omega }(u) - J'_{\omega }(u)h| \,\mathrm {d}\mathbb {P}(\omega )}{\Vert h \Vert _X} \\&\quad = \int _{\varOmega } \lim _{h \rightarrow 0} \frac{ |J_{\omega }(u+h) - J_{\omega }(u) - J'_{\omega }(u)h|}{\Vert h \Vert _X} \,\mathrm {d}\mathbb {P}(\omega ) = 0, \end{aligned} \end{aligned}$$

where the last equality follows by Assumption C.2(vii). Now consider the mapping $F: h \mapsto \int _{\varOmega } J_{\omega }'(u)h \,\mathrm {d}\mathbb {P}(\omega ).$ It is straightforward to show that this is a bounded and linear operator. Therefore, we use Assumption C.2(vi) to get

$$\begin{aligned}&\lim _{h \rightarrow 0} \frac{|\int _{\varOmega } J_{\omega }(u+h) \,\mathrm {d}\mathbb {P}(\omega ) - \int _{\varOmega }J_{\omega }(u) \,\mathrm {d}\mathbb {P}(\omega ) - F(h)| }{\Vert h \Vert _X} \\&\quad = \lim _{h \rightarrow 0} \frac{|\int _{\varOmega } (J_{\omega }(u+h) - J_{\omega }(u) - J'_{\omega }(u)h) \,\mathrm {d}\mathbb {P}(\omega )|}{\Vert h \Vert _X} = 0, \end{aligned}$$

where the second equality holds by the triangle inequality and and (C.3). Therefore *j* is Fréchet differentiable at *u* with derivative $F = \int _{\varOmega } J'_{\omega }(u) \,\mathrm {d}\mathbb {P}(\omega )$. $\square$

## Footnotes

1 The $\sigma$-algebra generated by a random variable $\beta :\varOmega \rightarrow \mathbb {R}$ is given by $\sigma (\beta ) = \{ \beta ^{-1}(B): B \in \mathcal {B}\}$, where $\mathcal {B}$ is the Borel $\sigma$-algebra on $\mathbb {R}$. Analogously, the $\sigma$-algebra generated by the set of random variables $\{ \beta _1, \dots , \beta _n\}$ is the smallest $\sigma$-algebra such that $\beta _i$ is measurable for all $i=1, \dots , n$.

## References

[1] Ali A, Ullmann E, Hinze M (2017). Multilevel Monte Carlo analysis for optimal control of elliptic PDEs with random coefficients. SIAM/ASA J. Uncertain. Quantif..

[2] Alnæs, M., Blechta, J., Hake, J., Johansson, A., Kehlet, B., Logg, A., Richardson, C., Ring, J., Rognes, M., Wells, G.: The FEniCS project version 1.5. Arch. of Numerical Software, 3(100) (2015)

[3] Barty K, Roy J, Strugarek C (2007). Hilbert-valued perturbed subgradient algorithms. Math. Oper. Res..

[4] Bauschke H, Combettes P (2011). Convex Analysis and Monotone Operator Theory in Hilbert Spaces.

[5] Beck, A.: First-Order Methods in Optimization, vol. 25. SIAM (2017)

[6] Bottou L (1998). Online learning and stochastic approximations. On-line Learn. Neural Netw..

[7] Bottou L, Curtis F, Nocedal J (2018). Optimization methods for large-scale machine learning. SIAM Rev..

[8] Brézis, H.: Opérateurs Maximaux Monotones et Semi-groupes de Contractions dans les Espaces de Hilbert. North-Holland Publishing Co., Amsterdam-London; American Elsevier Publishing Co., Inc., New York (1973)

[9] Cazenave T, Haraux A (1998). An Introduction to Semilinear Evolution Equations.

[10] Chen X, White H (2002). Asymptotic properties of some projection-based Robbins–Monro procedures in a Hilbert space. Stud. Nonlinear Dyn. Econom..

[11] Clarke F (1990). Optimization and Nonsmooth Analysis.

[12] Culioli J-C, Cohen G (1990). Decomposition/coordination algorithms in stochastic optimization. SIAM J. Control Optim..

[13] Davis D, Drusvyatskiy D, Kakade S, Lee J (2018). Stochastic subgradient method converges on tame functions. Found. Comput. Math..

[14] De Los Reyes J (2015). Numerical PDE-Constrained Optimization.

[15] Duchi J, Ruan F (2018). Stochastic methods for composite and weakly convex optimization problems. SIAM J. Optim..

[16] Duflo M (2013). Random Iterative Models.

[17] Ermoliev Y (1969). On the stochastic quasi-gradient method and stochastic quasi-Feyer sequences. Kibernetika.

[18] Evans, L.: Partial Differential Equations, volume Graduate Studies in Mathematics vol. 19. American Mathematical Society, Providence, R.I. (1998)

[19] Geiersbach C, Pflug G (2019). Projected stochastic gradients for convex constrained problems in Hilbert spaces. SIAM J. Optim..

[20] Geiersbach C, Wollner W (2020). A stochastic gradient method with mesh refinement for PDE-constrained optimization under uncertainty. SIAM/ASA J. Sci. Comput..

[21] Ghadimi S, Lan G, Zhang H (2016). Mini-batch stochastic approximation methods for nonconvex stochastic composite optimization. Math. Program..

[22] Goldstein L (1988). Minimizing noisy functionals in Hilbert space: an extension of the Kiefer–Wolfowitz procedure. J. Theor. Probab..

[23] Hinze M, Pinnau R, Ulbrich M, Ulbrich S (2009). Optimization with PDE Constraints.

[24] Keshavarzzadeh V, Fernandez F, Tortorelli D (2017). Topology optimization under uncertainty via non-intrusive polynomial chaos expansion. Comput. Methods Appl. Mech..

[25] Kiefer J, Wolfowitz J (1952). Stochastic estimation of the maximum of a regression function. Ann. Math. Stat..

[26] Kouri D, Surowiec T (2016). Risk-averse PDE-constrained optimization using the conditional value-at-risk. SIAM J. Optim..

[27] Kouri D, Surowiec T (2019). Risk-averse optimal control of semilinear elliptic PDEs. ESAIM Control Optim. Calc. Var..

[28] Kouri D, Heinkenschloss M, Ridzal D, Van Bloemen Waanders B (2013). A trust-region algorithm with adaptive stochastic collocation for PDE optimization under uncertainty. SIAM J. Sci. Comput..

[29] Kouri D (2014). A multilevel stochastic collocation algorithm for optimization of PDEs with uncertain coefficients. SIAM/ASA J. Uncertain. Quantif..

[30] Kunoth A, Schwab C (2016). Sparse adaptive tensor Galerkin approximations of stochastic PDE-constrained control problems. SIAM/ASA J. Uncertain. Quantif..

[31] Kupka I (1965). Counterexample to the Morse–Sard theorem in the case of infinite-dimensional manifolds. Proc. Am. Math. Soc..

[32] Kushner H, Yin G (2003). Stochastic Approximation and Recursive Algorithms and Applications.

[33] Kushner H, Clark D (1978). Stochastic Approximation Methods for Constrained and Unconstrained Systems.

[34] Lee H-C, Lee J (2013). A stochastic Galerkin method for stochastic control problems. Commun. Comput. Phys..

[35] Lei, J., Shanbhag, U.: A randomized block proximal variable sample-size stochastic gradient method for composite nonconvex stochastic optimization. arXiv preprint arXiv:1808.02543 (2018)

[36] Ljung L (1977). Analysis of recursive stochastic algorithms. IEEE Trans. Autom. Control.

[37] Martin, M., Krumscheid, S., Nobile, F.: Analysis of stochastic gradient methods for PDE-constrained optimal control problems with uncertain parameters. Technical report, École Polytechnique MATHICSE Institute of Mathematics (2018)

[38] Métivier M (2011). Semimartingales: A Course on Stochastic Processes.

[39] Nemirovski A, Juditsky A, Lan G, Shapiro A (2009). Robust stochastic approximation approach to stochastic programming. SIAM J. Optim..

[40] Nixdorf R (1984). An invariance principle for a finite dimensional stochastic approximation method in a Hilbert space. J. Multivar. Anal..

[41] Nouy A, Pled F (2018). A multiscale method for semi-linear elliptic equations with localized uncertainties and non-linearities. ESAIM: Math. Model. Numer..

[42] Okada N (1984). On the Banach–Saks property. Proc. Jpn. Acad. Ser. A Math. Sci..

[43] Pisier G (2016). Martingales in Banach Spaces.

[44] Reddi S, Sra S, Poczos B, Smola A (2016). Proximal stochastic methods for nonsmooth nonconvex finite-sum optimization. Adv. Neural Inf. Process. Syst..

[45] Robbins H, Monro S (1951). A stochastic approximation method. Ann. Math. Stat..

[46] Robbins H, Siegmund D (1971). A convergence theorem for non negative almost supermartingales and some applications. Optimizing Methods in Statistics.

[47] Rosseel E, Wells G (2012). Optimal control with stochastic PDE constraints and uncertain controls. Comput. Methods Appl. Mech. Eng..

[48] Ruszczynski A, Syski W (1983). Stochastic approximation method with gradient averaging for unconstrained problems. IEEE Trans. Autom. Control.

[49] Shapiro A, Wardi Y (1996). Convergence analysis of stochastic algorithms. Math. Oper. Res..

[50] Smale S (2000). An infinite dimensional version of Sard’s theorem. The Collected Papers of Stephen Smale.

[51] Tiesler H, Kirby R, Xiu D, Preusser T (2012). Stochastic collocation for optimal control problems with stochastic PDE constraints. SIAM J. Control Optim..

[52] Tröltzsch, F.: Optimale Steuerung partieller Differentialgleichungen. Vieweg + Teubner, 2nd edition (2009)

[53] Uryasev SP (1992). A stochastic quasigradient algorithm with variable metric. Ann. Oper. Res..

[54] Van Barel A, Vandewalle S (2019). Robust optimization of PDE constrained systems using a multilevel Monte Carlo method. SIAM/ASA J. Uncertain. Quantif..

[55] Venter JH (1966). On Dvoretzky stochastic approximation theorems. Ann. Math. Stat..

[56] Wardi Y (1989). A stochastic algorithm using one sample point per iteration and diminishing stepsizes. J. Optim. Theory Appl..

[57] Williams D (1991). Probability with Martingales.

[58] Yin G, Zhu YM (1990). On $$H$$-valued Robbins–Monro processes. J. Multivar. Anal..

